# Genetic analysis of disease resilience of wean-to-finish pigs under a natural disease challenge model using reaction norms

**DOI:** 10.1186/s12711-022-00702-0

**Published:** 2022-02-08

**Authors:** Jian Cheng, KyuSang Lim, Austin M. Putz, Anna Wolc, John C. S. Harding, Michael K. Dyck, Frederic Fortin, Graham S. Plastow, PigGen Canada, Jack C. M. Dekkers

**Affiliations:** 1 Department of Animal Science, Iowa State University, Ames, IA 50011 USA; 2 Swine Business Unit, Hendrix Genetics, Boxmeer, 5831 CK The Netherlands; 3 Hy-Line International, Dallas Center, IA 50063 USA; 4 Department of Large Animal Clinical Sciences, University of Saskatchewan, Saskatoon, SK S7N 5B4 Canada; 5 Department of Agricultural, Food and Nutritional Science, University of Alberta, Edmonton, AB T6G 2R3 Canada; 6 Centre de Développement du Porc du Québec Inc., Québec City, G1V 4M6 Canada; 7 PigGen Canada Research Consortium, Guelph, ON N1H4G8 Canada

## Abstract

**Background:**

Disease resilience is the ability to maintain performance across environments with different disease challenge loads (CL). A reaction norm describes the phenotypes that a genotype can produce across a range of environments and can be implemented using random regression models. The objectives of this study were to: (1) develop measures of CL using growth rate and clinical disease data recorded under a natural polymicrobial disease challenge model; and (2) quantify genetic variation in disease resilience using reaction norm models.

**Methods:**

Different CL were derived from contemporary group effect estimates for average daily gain (ADG) and clinical disease phenotypes, including medical treatment rate (TRT), mortality rate, and subjective health scores. Resulting CL were then used as environmental covariates in reaction norm analyses of ADG and TRT in the challenge nursery and finisher, and compared using model loglikelihoods and estimates of genetic variance associated with CL. Linear and cubic spline reaction norm models were compared based on goodness-of-fit and with multi-variate analyses, for which phenotypes were separated into three traits based on low, medium, or high CL.

**Results:**

Based on model likelihoods and estimates of genetic variance explained by the reaction norm, the best CL for ADG in the nursery was based on early ADG in the finisher, while the CL derived from clinical disease traits across the nursery and finisher was best for ADG in the finisher and for TRT in the nursery and across the nursery and finisher. With increasing CL, estimates of heritability for nursery and finisher ADG initially decreased, then increased, while estimates for TRT generally increased with CL. Genetic correlations for ADG and TRT were low between high versus low CL, but high for close CL. Linear reaction norm models fitted the data significantly better than the standard genetic model without genetic slopes, while the cubic spline model fitted the data significantly better than the linear reaction norm model for most traits. Reaction norm models also fitted the data better than multi-variate models.

**Conclusions:**

Reaction norm models identified genotype-by-environment interactions related to disease CL. Results can be used to select more resilient animals across different levels of CL, high-performance animals at a given CL, or a combination of these.

**Supplementary Information:**

The online version contains supplementary material available at 10.1186/s12711-022-00702-0.

## Background

In swine breeding, selection for economically important traits is usually based on phenotypes recorded on purebred animals in nucleus farms with a high health status. However, the breeding goal is to improve the performance of crossbred animals raised in commercial farms, which typically have a lower level of biosecurity. The effectiveness of these purebred selection programs depends on the genetic correlations of trait phenotypes collected on purebred animals in the nucleus environment with phenotypes expressed for those same traits in crossbreds in commercial environments. Wientjes and Calus [1] reviewed published estimates of purebred–crossbred genetic correlations in pigs and found estimates that averaged 0.63, with only 50% of the estimates > 0.45 (up to 0.87). They also found that genotype-by-environment interaction is one of the main factors that contributes to these low genetic correlations.

Disease resilience is defined as the ability of an animal to maintain performance across environments with different disease challenge loads (CL) [2, 3]. Bisset and Morris [3] pointed out that disease resilience captures both resistance and tolerance, where disease resistance is defined as the ability of an animal to prevent infection when exposed to a pathogen or to limit replication of the pathogen when infected [4, 5], and disease tolerance is defined as the ability of an animal to maintain performance at a given level of infection or pathogen load [5]. In contrast to resistance and tolerance, evaluation of disease resilience does not require knowledge of pathogen burden [6], which is often difficult to obtain, but uses measures of performance in environments with different CL. Albers et al. [2] used the reduction in growth rate due to infection to measure disease resilience in sheep.

A reaction norm describes the phenotypes that a genotype can produce across a range of environments [7, 8]; resilient animals tend to maintain the same performance level across different environments. A random regression model (RRM) represents a parsimonious covariance structure that can model breeding values for a phenotype as a function of one or more continuous variables [9, 10] and, thus, can implement reaction norm models, as demonstrated in several studies [11, 12]. Kause [13] demonstrated that genetic variance in tolerance can be estimated as genetic variance in regression slopes of performance on pathogen burden, where the slope represents the ability of an animal to cope with different CL. Resilience can be analyzed as a reaction norm with the animal’s performance regressed on a measure of CL, which does not require measures of pathogen burden but can be derived using performance for a contemporary group and/or clinical records [14–16]. Mathur et al. [17] proposed a method to estimate the CL using sow reproduction records in natural outbreaks of porcine reproductive and respiratory syndrome (PRRS), based on the assumption that the reduction in weekly reproductive output in a farm during an outbreak is proportional to the magnitude of the challenge. This method was also used by Rashidi et al. [14] to identify healthy and diseased time periods in sow farms in which PRRS was endemic and to evaluate genetic variation in resilience of sows to PRRS CL using reaction norm models. Guy et al. [15] used reaction norm models to assess resilience of pigs based on different environmental descriptors derived from estimates of the contemporary group effect for different phenotypes such as growth, feed intake, and carcass traits, and found that the use of different phenotypes differed in their ability to detect genetic variation in reaction norm slopes.

Recently, Putz et al. [18] and Cheng et al. [19] described a natural disease challenge model for wean-to-finish pigs, in which clinical disease phenotypes were recorded on batches of 60 or 75 pigs, including medical treatment rates, mortality rates, and subjective health scores. These clinical disease traits are a direct reflection of the severity of the disease challenge. To our knowledge, few studies have used clinical disease phenotypes as CL or as environment factors to study disease resilience. Guy et al. [16] used medication treatment records to define the CL in pigs for studying disease resilience, but medications were mainly associated with tail biting, rather than disease. In dairy cattle, milk somatic cell count was used as an environmental descriptor to identify genotype-by-environment interactions for milk protein yield [20], but this is limited to milking animals and cannot be widely applied to all livestock. The objectives of this study were to: (1) develop different CL using growth rate and clinical disease phenotypes collected under the natural polymicrobial disease challenge model described by Putz et al. [18] and Cheng et al. [19]; and (2) evaluate genetic variation in disease resilience using reaction norm models based on the developed CL.

## Methods

This study was carried out in accordance with the Canadian Council on Animal Care guidelines (CCAC; https://www.ccac.ca/en/certification/about-certification). The protocol was approved by the Protection Committee of the Centre de Recherche en Sciences Animales de Deschambault (CRSAD) and the Animal Care and Use Committee at the University of Alberta (AUP00002227). The project was fully overseen by the Centre de Développement du Porc du Québec (CDPQ) in Québec, Canada, and its herd veterinarian together with project veterinarians.

### Phenotypes and genotypes

**Fig. 1:**
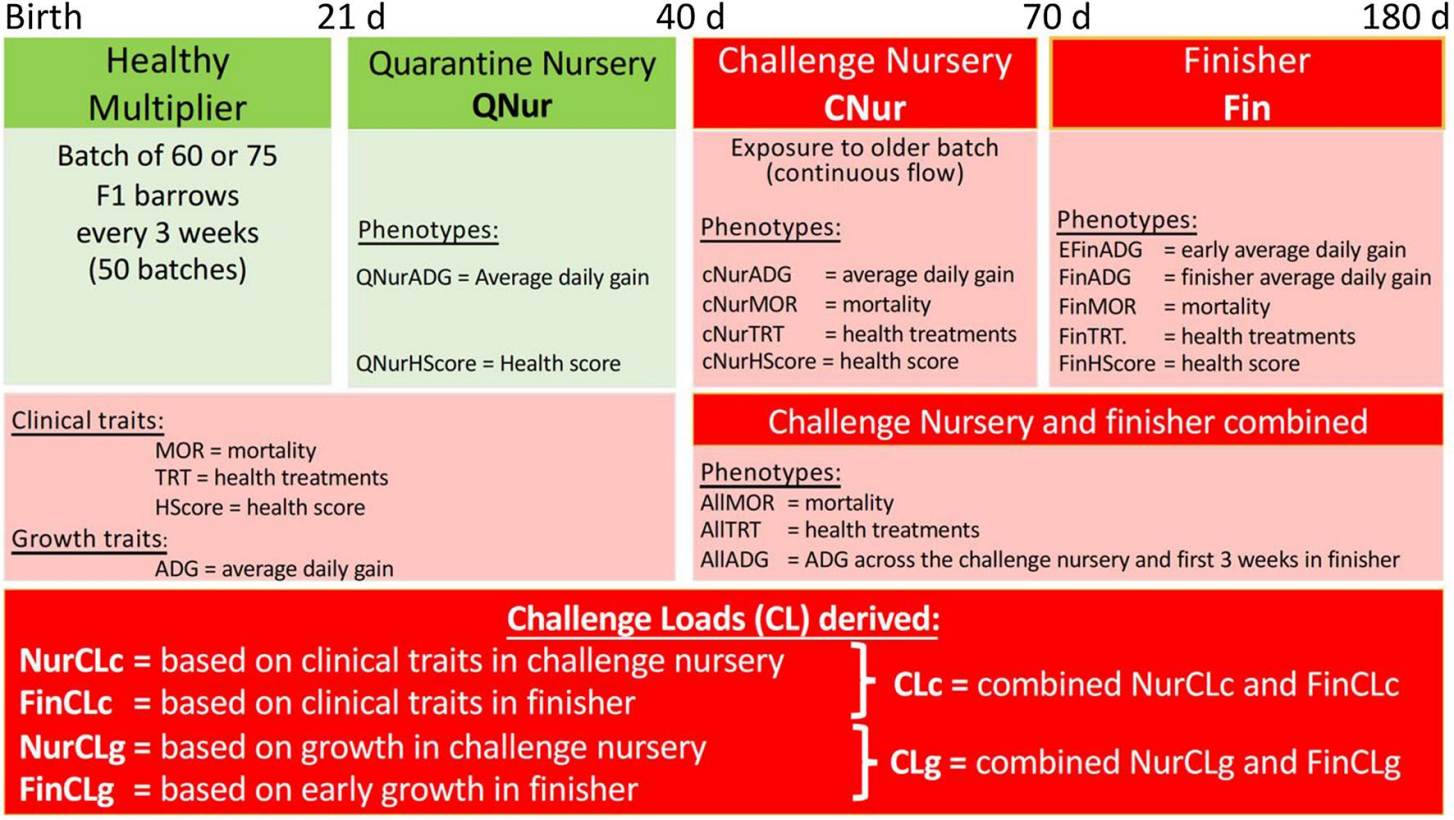
Natural disease challenge protocol, phenotypes, and challenge loads. Green = healthy. Red = challenged

All data were collected by trained research staff from CDPQ, using a natural disease challenge wean-to-finish barn that was established in 2015 by bringing naturally infected animals into a late nursery and finish barn (Fig. 1). The natural challenge included various viral and bacterial pathogens that are common in the industry and was maintained by entering batches of 60 or 75 healthy nursery pigs every three weeks in a continuous flow system (see Putz et al. [18] for details). For each batch, weaned Large White x Landrace barrows were provided by one of seven breeding companies, with each company providing one batch for each cycle, for a total of seven cycles. The natural challenge protocol consisted of three phases: (1) quarantine nursery (19 days on average beginning at 3 weeks of age), (2) challenge nursery (27 days on average), and (3) finishing phase (100 days on average). Average group sizes in the three phases were 4.3, 7.2, and 10.7 pigs per pen, respectively, and pigs were re-grouped when moved between phases. The objective of the challenge was to mimic that of high disease pressure in a commercial farm to maximize the expression of genetic differences for disease resilience. In total, phenotypes and genotypes on 3205 pigs were available, as described by Cheng et al. [19]. Phenotypes included average daily gain in the challenge nursery (cNurADG) and in the finisher (FinADG), medical treatment rates in the challenge nursery (cNurTRT), in the finisher (FinTRT), and across the nursery and the finisher (AllTRT), mortality rates in the challenge nursery (cNurMOR), in the finisher (FinMOR), and across the nursery and the finisher (AllMOR), and subjective health scores in the challenge nursery (cNurHScore) and in the finisher (FinHScore). Phenotypes for ADG were derived as the regression of body weight on age, separately for the challenge nursery and the finisher. ADG in the finisher was also computed using only body weights from the first three weeks in the finisher, because most pigs had recovered from the disease challenge later in the finishing phase. This ADG will be referred to as early finisher ADG (EFinADG). Phenotypes for ADG across the challenge nursery and the first three weeks in the finisher were also derived and will be referred to as AllADG. Health scores were assigned on a 1 to 5 scale by highly qualified research personnel based on clinical signs, regardless of the cause of the symptoms: 1 = severe clinical signs with loss of weight and strength and 5 = in perfect health. Treatment rates were standardized by multiplying the number of treatments a pig received by the ratio of the average length of the corresponding phase and the number of days the pig spent in that phase. Mortality was recorded as 0 = survived and 1 = died. Details on these phenotypes are in Cheng et al. [19]. For treatment and growth rates in the finisher, data from pigs that died in the finisher were included in the analyses after imputing the truncated records to complete phenotypes, as described by Cheng et al. [19].

All animals were genotyped with the 650 k Affymetrix Axiom Porcine Genotyping Array for 658,692 single nucleotide polymorphisms (SNPs) by Delta Genomics (Edmonton AB, Canada). Raw Affymetrix SNP data were processed by Delta Genomics, separately for each cycle, with the Axiom Analysis Suite, using all defaults. The 435,172 SNPs that passed quality control (minor allele frequency > 0.05; call rate for marker > 0.90; call rate for individual > 0.90) for 3205 pigs, were used for analysis.

### Disease challenge load

To derive a quantitative measure of the CL that pigs faced for use as an environmental covariate in reaction norm models for average daily gain (ADG) and medical treatment rate (TRT), it was assumed that the CL differed between batches and between pens within a batch, in both the challenge nursery and the finisher. The CL by batch and pen was derived from estimates of the environmental effects associated with batch and pen for either ADG or for the clinical disease traits TRT (including individual and batch treatments), MOR, and health scores (HScore). Estimates of the environmental effects associated with batch and pen for these traits were obtained from the following model, as used by Cheng et al. [19], for ADG, TRT, MOR, and HScore, separately for the challenge nursery and the finisher:

1 $${y}_{ijkl}=\,Batc{h}_{i}+Die{d}_{ijkl}+b*Ag{e}_{ijkl}+ Pe{n}_{j}+ {u}_{ijkl}+ litte{r}_{ijk}+{e}_{ijkl},$$

where ${y}_{ijkl}$ is the phenotype of pig $l$ of batch $i$ in pen $j$ from litter $ijk$ for ADG, TRT, MOR, or HScore in the challenge nursery or the finisher; $Batc{h}_{i}$ is the fixed batch effect ($i$ = 1, …, 50); $Die{d}_{ijkl}$ is the fixed effect of the pig dying during the corresponding phase, which was fitted only for cNurADG, FinADG, and AllTRT; $Ag{e}_{ijkl}$ is the covariate of age when the pig entered the quarantine nursery, with regression coefficient $b$; $Pe{n}_{j}$ is the random effect of pen within batch (437 unique pens in the challenge nursery and 262 in the finisher), with vector $\mathbf{P}\mathbf{e}\mathbf{n} \sim N(0, {\mathbf{I}\sigma }_{P}^{2})$*,* where ${\sigma }_{Pl}^{2}$ is the pen variance and $\mathbf{I}$ an identity matrix of appropriate size; ${u}_{ijkl}$ is the random additive genetic effect, with vector $\mathbf{u}\sim N(0, \mathbf{G}{\sigma }_{A}^{2}$*)*, where $\mathbf{G}$ is the genomic relationship matrix, computed as described by Cheng et al. [19], and ${\sigma }_{A}^{2}$ is the additive genetic variance; $litte{r}_{ijk}$ is the common environmental effect associated with litter mates (1119 litters), with vector $\mathbf{l}\mathbf{i}\mathbf{t}\mathbf{t}\mathbf{e}\mathbf{r}\sim N(0, \mathbf{I}{\sigma }_{k}^{2}$*),* where ${\sigma }_{k}^{2}$ is the litter environmental variance; and ${e}_{ijkl}$ is the residual effect, with vector $\mathbf{e}\sim N(0, {\mathbf{I}\sigma }_{e}^{2}$*)*, where ${\sigma }_{e}^{2}$ is the residual variance.

Based on the resulting estimates of batch and pen effects, several alternate CL were derived for each pen within batch. Challenge loads based on ADG in the challenge nursery (NurCLg) were derived as the sum of batch and pen estimates obtained from analysis of cNurADG with model (1), including ADG of pigs that died in the nursery. Similarly, CL based on ADG in the finisher (FinCLg) were derived as the sum of batch and pen estimates from analysis of EFinADG with model (1), using data on pigs that survived at least three weeks in the finisher. Here, EFinADG was used because ADG based on all body weight data in the finisher may not reflect the disease challenge pigs experienced in the finisher. To obtain a combined CL across the challenge nursery and the finisher (CLg), NurCLg and FinCLg were weighted as:

2 $$CLg=0.35*NurCLg+0.65*FinCLg.$$

The weights in model (2) were derived as the regression coefficients of NurCLg and FinCLg on ADG across the challenge nursery and the first three weeks in the finisher (AllADG), which were estimated by adding NurCLg and FinCLg as covariates to model (1) for analysis of phenotypes for AllADG that were pre-adjusted using estimates of batch and pen effects from model (1).

Challenge loads based on clinical disease traits (CLc) for each pen within batch were estimated as a quadratic function of the estimates of batch and pen effects from model (1) for TRT, MOR, and HScore, separately for the challenge nursery and the finisher (NurCLc and FinCLc, respectively):

3 $$\begin{array}{ll}NurCLc={\sum }_{m=1}^{2}{w}_{1m}NurTR{T}_{batch}^{m}\\ \qquad \qquad\,\, +\,{\sum }_{m=1}^{2}{w}_{2m}NurTR{T}_{pen}^{m} +{\sum }_{m=1}^{2}{w}_{3m}NurMO{R}_{batch}^{m}\\\qquad \qquad\,\,+\,{\sum }_{m=1}^{2}{w}_{4m}NurMO{R}_{pen}^{m}+ {\sum }_{m=1}^{2}{w}_{5m}NurHScor{e}_{batch}^{m}+ {\sum }_{m=1}^{2}{w}_{6m}NurHScor{e}_{pen}^{m},\end{array}$$

where, $m$ =1 and 2 stands for linear and quadratic terms, respectively, ${w}_{1m}$, ${w}_{2m}$, ${w}_{3m}$, ${w}_{4m}$, ${w}_{5m}$, and ${w}_{6m}$ are weighting factors, $NurTR{T}_{batch}$, $NurMO{R}_{batch}$, and $NurHScor{e}_{batch}$ are estimates of batch effects for the corresponding traits from model (1), and $NurTR{T}_{pen}$, $NurMO{R}_{pen}$, and $NurHScor{e}_{pen}$ are estimates of pen effects for the corresponding traits from model (1). A quadratic function was used to accommodate potential non-linear relationships of pen and batch effects with challenge load. Similarly, FinCLc was estimated as a function of the estimates of batch and pen effects for FinTRT, FinMOR, and FinHScore. The weighting factors in model (3) were derived on the assumption that the contributions of TRT, MOR, and HScore to the challenge load are proportional to their impacts on ADG, i.e. cNurADG and EFinADG. On this premise, the weighting factors were derived by regressing the sum of estimates of batch and pen effects from model (1) for cNurADG and EFinADG, respectively, on the estimates of batch and pen effects using model (3). A stepwise model selection strategy by backward elimination was performed to select the batch and pen effect estimates that significantly affected NurCLc and FinCLc (P-value < 0.05). The resulting equations to estimate NurCLc and FinCLc were:

4 $$\begin{array}{ll}NurCLc=1.43-0.31*NurTR{T}_{batch}+5.95*NurMO{R}_{batch}\\ \quad\qquad\quad -\, 5.19*NurMO{R}_{batch}^{2}-10.01*NurHScor{e}_{batch}-4.99*NurHScor{e}_{batch}^{2}\\ \quad\qquad\quad+\, 0.46*NurMO{R}_{pen}-1.28*NurMO{R}_{pen}^{2}\\\quad\qquad\quad-\,1.40*NurHScor{e}_{pen}-1.29*NurHScor{e}_{pen}^{2},\end{array}$$

5 $$\begin{array}{ll}FinCLc=0.03+0.04*FinTR{T}_{batch}+0.62*FinMO{R}_{batch}\\\qquad \qquad-\,0.15*FinHScor{e}_{batch}-0.33*FinHScor{e}_{pen}\\\qquad \qquad -\,0.28*FinHScor{e}_{pen}^{2}.\end{array}$$

To derive a combined CL across the challenge nursery and finisher, CLc was weighted by NurCLc and FinCLc, as for model (2), with weighting factors derived by adding NurCLc and FinCLc to model (1) as covariates for phenotypes for AllADG that were pre-adjusted using estimates of batch and pen effect from model (1). Based on the resulting regression coefficient estimates, CLc was weighted as:

6 $$CLc=0.12*NurCLc+0.88*FinCLc.$$

All CL were standardized to a mean of zero and a standard deviation (SD) of one for use in further analyses.

To determine to what extent each of the derived CL was affected by batch versus pen within batch effects, the proportion of variance explained by batch was estimated for each CL by fitting a model with batch as random effect the derived CL for each pen. In this model, the residual variance is equal to the variance of pen within batch because the CL was derived from estimates of batch and pen within batch from model (1).

### Reaction norm models

A two-step approach was used for reaction norm analyses of cNurADG, FinADG, cNurTRT, and AllTRT, using each of the six derived CL (CLc, NurCLc, FinCLc, CLg, NurCLg, and FinCLg). FinTRT was not analyzed because of its close to zero estimate of heritability [19]. First, all phenotypes were pre-adjusted for the effects of batch and pen using model (1). Then, either a linear reaction norm model or a cubic spline reaction norm model was fitted to the pre-adjusted phenotypes. Compared to the linear reaction model, the cubic spline model is more flexible and does not assume a linear relationship between the response trait and the environmental variable (CL in this study). A quadratic reaction norm model was also attempted but had convergence issues for most traits and was, therefore, not further pursued.

The following model was used for the linear reaction norm analyses:

7 $${y}_{kl}=\,{Died}_{kl}+b*Ag{e}_{kl}+{\beta *CL}_{kl}+{u}_{kl0}{+ u}_{kl1}*C{L}_{kl}+litte{r}_{k}+{e}_{kl,}$$

where ${y}_{kl}$ is the phenotype of pig $kl$ for ADG or TRT; $Ag{e}_{kl}$ and $litte{r}_{k}$ are the same as in model (1), except that $litte{r}_{k}$ was only fitted for cNurADG and cNurTRT because FinADG and AllTRT had small litter effects; ${Died}_{kl}$ indicates whether the pig died or not during the corresponding phase; ${\beta *CL}_{kl}$ is the fixed covariate effect for CL, with regression coefficient $\beta$; ${u}_{kl0}$ and ${u}_{kl1}$ are the random additive genetic effects for the intercept and slope, respectively, for the reaction norm for animal $kl$. The variance–covariance structure for the vectors of random regression coefficients was:

$$Var\left[\begin{array}{c}{\mathbf{u}}_{kl0}\\ {\mathbf{u}}_{kl1}\end{array}\right]\sim N\left(0, \mathbf{G} \otimes \left[\begin{array}{cc}{\sigma }_{0}^{2}& {\sigma }_{\mathrm{0,1}}\\ {\sigma }_{\mathrm{1,0}}& {\sigma }_{1}^{2}\end{array}\right]\right),$$

where ${\sigma }_{0}^{2}\, and\, {\sigma }_{1}^{2}$ are the genetic variances for the intercept and slope, respectively, and ${\sigma }_{\mathrm{0,1}}$ is the corresponding genetic covariance.

The cubic spline reaction norm model consisted of a series of piecewise cubic polynomials between defined knots (breakpoints) for the CL that were constrained such that the cubic spline function and its first two derivatives are continuous at the knots [21]. The cubic spline reaction norm model that was fitted here used knots at the minimum, medium, and maximum CL, respectively, following de Groot et al. [22] (a model with four knots was also attempted but resulted in a poorer fit), and can be described as:

8 $${y}_{kl}=\,{Died}_{kl}+b*Ag{e}_{kl}+{\beta *CL}_{kl}{+ s}_{kl0}*C{L}_{kl}+{u}_{kl0}{+ u}_{kl1}*C{L}_{kl} {+ s}_{kl1}*C{L}_{kl} + litte{r}_{k}+{e}_{kl},$$

where all terms are as described in model (7), except that the terms ${s}_{kl0}*C{L}_{kl}$ and ${s}_{kl1}*C{L}_{kl}$ were added based on the incorporation of cubic splines in the mixed model equations described by de Groot et al. [22], where ${s}_{kl0}$ is a fixed cubic spline effect and ${s}_{kl1}$ is the random additive genetic spline effect for animal $kl$. The variance–covariance structure for the vectors of additive genetic effects for this model was:

$$Var\left[\begin{array}{c}{\mathbf{u}}_{kl0}\\ {\mathbf{u}}_{kl1}\\ {\mathbf{s}}_{kl1}\end{array}\right]\sim N\left(0, \mathbf{G} \otimes \left[\begin{array}{ccc}{\sigma }_{0}^{2}& {\sigma }_{\mathrm{0,1}}& 0\\ {\sigma }_{\mathrm{1,0}}&{\sigma }_{1}^{2}& 0\\ 0& 0& {\sigma }_{as}^{2}\end{array}\right]\right),$$

where ${\sigma }_{0}^{2}$, ${\sigma }_{1}^{2}$, and ${\sigma }_{\mathrm{1,0}}$ are as described for model (7) and ${\sigma }_{as}^{2}$ is the genetic variance of the random spline effects. Both the linear and cubic spline reaction norm models (7) and (8) allowed for heterogeneous environmental variances for three classes of CL, i.e., low, intermediate, and high CL, with approximately 1000 animals in each CL class. Thus, phenotypes of animals that were in the same CL class were assumed to have the same environmental variance. All analyses were implemented in ASReml 4.0 [23].

The matrix of genetic variances and covariances for the analyzed trait for $n$ levels of CL, as generated by ASReml, is equal to $\mathbf{T}\mathbf{V}\mathbf{T}\mathbf{^{\prime}}$ for model (7) and $\mathbf{T}\mathbf{V}\mathbf{T}\mathbf{^{\prime}}+\boldsymbol{ }\mathbf{z}\mathbf{z}\mathbf{^{\prime}}{\sigma }_{as}^{2}$ for model (8) [22], where $\mathbf{T}$ is an *n* $\times$ 2 matrix for the intercept and slope corresponding to the $n$ CL levels, $$\mathbf{V}=\mathbf{G} \otimes \left[\begin{array}{cc}{\sigma }_{0}^{2}& {\sigma }_{\mathrm{0,1}}\\ {\sigma }_{\mathrm{1,0}}& {\sigma }_{1}^{2}\end{array}\right]$$, and $\mathbf{z}$ is an *n* $\times$ 1 vector of the animal genetic spline coefficients for the $n$ CL levels. Estimates of genetic variances and co-variances of the trait for and between different CL levelscwere derived by replacing the variance and co-variance components in these equations by their estimates.

Two comparisons were used to determine which CL captured genetic effects associated with the reaction norms the best. For the first comparison, all six CL were simultaneously fitted as fixed covariates in the linear and cubic spline reaction norm models but only one CL was fitted for the genetic slope effect, and the resulting model loglikelihoods and estimates of genetic variance associated with the reaction norm were compared between the different CL. In this comparison, the fixed spline function ${s}_{kl0}*C{L}_{kl}$ was excluded from the cubic spline reaction norm model such that the same fixed effects were included for comparison of model likelihoods. The main purpose of this comparison was to compare the CL for a given model (i.e. the linear or the cubic spline reaction norm model). In the second comparison, estimates of genetic variances for the reaction norm slopes from models (7) and (8) with only the corresponding CL fitted as fixed covariate were compared. This comparison was also used to compare the goodness-of-fit of the linear and cubic reaction norm models.

The linear reaction norm model using one of the six CL was also compared to a standard mixed linear model for the pre-adjusted phenotypes, which was the same as model (7) but without the random slope term. The cubic spline reaction norm model was compared to the linear reaction norm model (7) for a given CL but with ${s}_{kl0}*C{L}_{kl}$ added as a fixed effect to the latter, such that the likelihoods from the two models were comparable. A likelihood ratio test was used to evaluate the goodness-of-fit when comparing two models, based on a Chi-square distribution with degrees of freedom equal to the difference in dimensionality of the parameters of the two models [24].

Model (7) without the random slope term was also used for a three-trait multi-variate analysis, where the phenotype in each of the three classes of CL (low, medium, and high) that were used to define heterogenous residual variances, was considered a different genetic trait. The multi-variate model does not assume a specific functional relationship of additive genetic effects with CL and, therefore, enables one to assess whether parameter estimates from the reaction norm models may be ‘biased’ by the specific functional relationship that is fitted in the linear or cubic reaction norm models. A disadvantage of a multi-variate model, however, is that the continuous variable CL is broken up into intervals, within which genetic parameters are assumed to be constant. Estimates of genetic parameters from the multi-variate model were compared with corresponding estimates obtained from the linear and cubic spline reaction norm models at the mean CL of the low, intermediate, and high CL classes. Goodness-of-fit of the multi-variate and the linear and cubic reaction norm models were also compared based on the Akaike information criterion (AIC).

## Results

### Phenotypic trends for clinical disease traits and growth rate

**Fig. 2:**
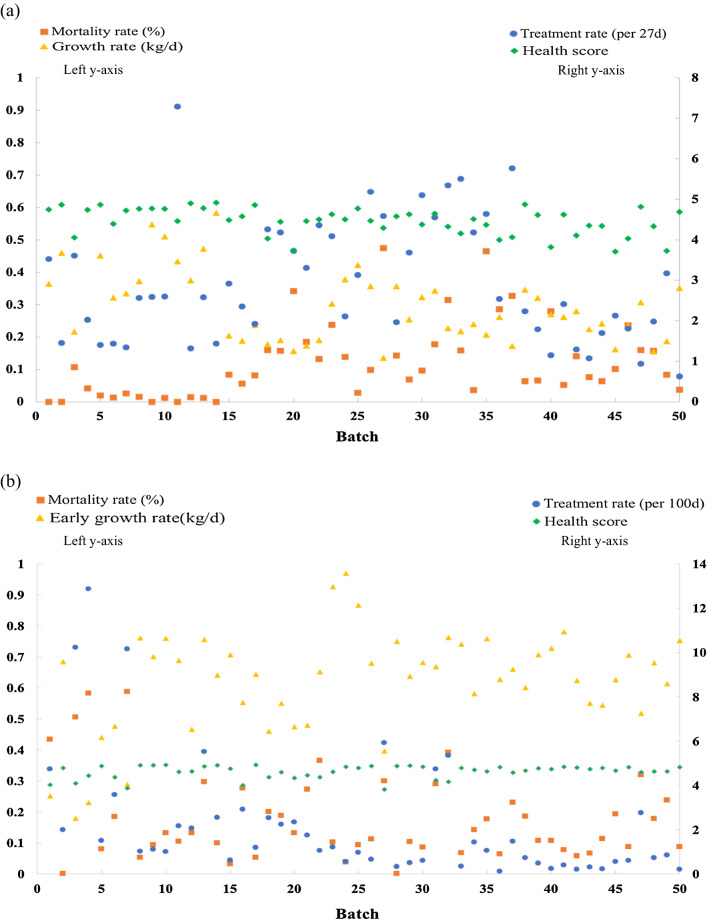
Treatment and mortality rates and average health scores and growth rates by batch in the challenge nursery (**a**) and in the finisher (**b**)

Average treatment and mortality rates, subjective health scores, and growth rates by batch in the challenge nursery and finisher are shown in Fig. 2. In the challenge nursery, in general, the average treatment rate increased and peaked at around batch 35 and then decreased. Mortality rate followed a similar trend, with relatively higher mortality rates for batches 20–40. Average health scores and growth rates were both higher in the early batches and then decreased, which was in contrast to the observed trends for treatment rate and mortality, except for the final batches. In the finisher, generally, higher treatment and mortality rates were always associated with lower average health scores and growth rates. For example, treatment and mortality rates were higher in the early batches, while average health scores and growth rates were relatively lower in the early batches.

Trends in average clinical disease phenotypes by batch were not consistent across the two phases (Fig. 2). Treatment and mortality rates were lower in the early batches in the challenge nursery but relatively higher in the finisher. Treatment and mortality rates were high in the middle batches in the challenge nursery but low in the finisher. In the later batches, treatment rate and average health scores had much greater variation in the challenge nursery than in the finisher.

### Challenge loads

**Fig. 3:**
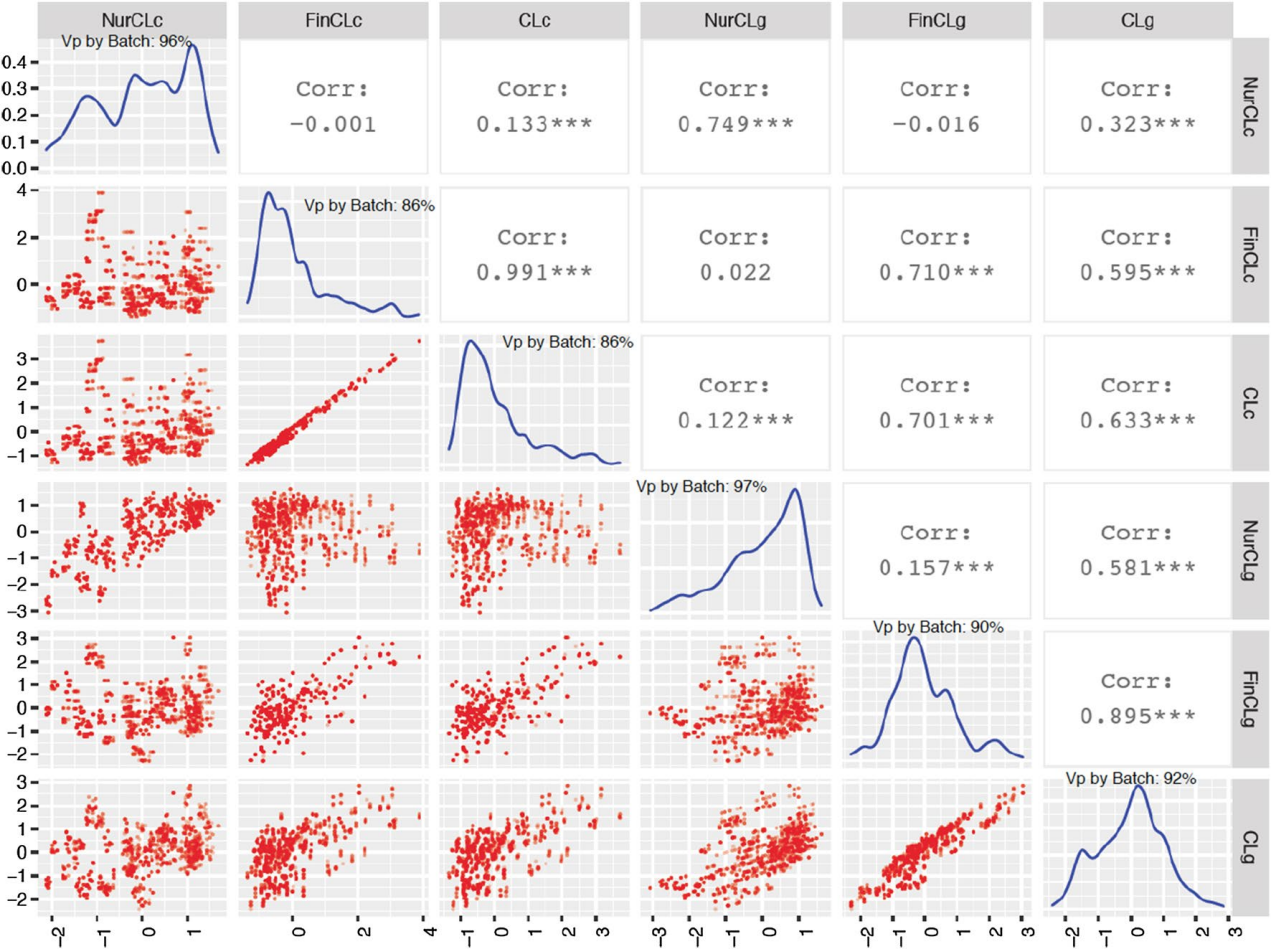
Distributions and relationships of challenge loads by pen within batch derived from the growth and clinical disease phenotypes. Vp by Batch: proportion of phenotypic variance explained by Batch for each CL; NurCLc = challenge load derived from the clinical disease traits in the challenge nursery; FinCLc = challenge load derived from the clinical disease traits in early finisher; CLc = weighted challenge load of NurCLc and FinCLc; NurCLg = challenge load derived from the growth rate in the challenge nursery; FinCLg = challenge load derived from the growth rate in early finisher; CLg = weighted challenge load of NurCLg and FinCLg

Distributions of the six standardized (mean zero and SD equal to 1) CL and their relationships are shown in Fig. 3. Large variation was observed for each CL but 86 to 97% of this variation was attributed to batch effects. Most of the CL were skewed, e.g. CLc was skewed to the right. In the remainder, results will focus on the 95% highest density interval (HDI) for each CL, which provides more reliable results. CLc was highly correlated with FinCLc (0.99) and the same was true for CLg with FinCLg (0.90), which was because CLc and CLg were heavily weighted by FinCLc and FinCLg, respectively. The CL derived from the clinical disease traits and from ADG for a given phase were moderately correlated (0.75 between NurCLg and NurCLc, 0.71 between FinCLg and FinCLc, and 0.63 between CLg and CLc). Finally, FinCLg was moderately correlated with CLc (0.70).

### Comparison of CL in reaction norm analyses

Tables 1 and 2 show the loglikelihoods and slope genetic variances from the linear and cubic spline reaction norm models when all six CL were fitted as fixed covariates and one CL as the random reaction norm. In general, estimates of the slope genetic variance based on the cubic spline model were slightly higher than those based on the linear model. For a given trait, the ranking of CL was similar when based on loglikelihood or on estimates of the slope genetic variance and when based on the linear or the cubic spline reaction norm model. However, the CL that was best based on the loglikelihood differed between traits, for both the linear and cubic reaction norm models. The same was true when using the slope genetic variance to identify the best CL.

**Table 1:** Comparison of loglikelihoods for the linear and cubic reaction norm models for analysis of average daily gain (ADG) and treatment rate (TRT) in the challenge nursery (cNur), finisher (Fin) and across the nursery and finisher (All) for six challenge loads based on clinical disease traits or growth rate

Trait	Loglikelihood using clinical disease traits			Loglikelihood using growth rate		
	NurCLc	FinCLc	CLc	NurCLg	FinCLg	CLg
Linear RN						
cNurADG	4718.26	4727.34	4725.71	4715.68	*4744.44*	4736.78
FinADG	*3844.07*	3802.61	3797.06	3815.21	NA	3806.87
cNurTRT	− 1802.20	*− 1753.47*	− 1761.52	NA	− 1797.27	− 1792.54
AllTRT	− 2166.53	− 2147.13	− 2149.99	− 2169.70	− 2150.46	*− 2146.40*
Cubic RN						
cNurADG	4720.07	4728.97	4730.27	4719.09	*4745.39*	4737.24
FinADG	*3844.07*	3802.84	3800.26	3820.95	3817.12	3815.47
cNurTRT	− 1802.20	*− 1752.64*	− 1758.89	− 1797.79	− 1797.27	− 1792.54
AllTRT	− 2166.53	*− 2143.02*	− 2143.89	− 2169.70	− 2150.46	− 2146.40

All six CL were fitted as fixed effects in the linear and cubic reaction norm model, but only one CL was fitted for the random genetic reaction norm. cNurADG: growth rate in the challenge nursery; FinADG: growth rate in the finisher; cNurTRT: medical treatment rate in the challenge nursery; AllTRT: medical treatment rate across the challenge nursery and finisher; NurCLc: challenge load derived from the clinical disease traits in the challenge nursery; FinCLc: challenge load derived from the clinical disease traits in early finisher; CLc: weighted challenge load of NurCLc and FinCLc; NurCLg: challenge load derived from the growth rate in the challenge nursery; FinCLg: challenge load derived from the growth rate in early finisher; CLg: weighted challenge load of NurCLg and FinCLg; figures in italic characters indicate the greatest loglikelihood for that trait

**Table 2:** Comparison of slope genetic variances for the linear and cubic reaction norm models for analysis of average daily gain (ADG, g) and treatment rate (TRT) in the challenge nursery (cNur), finisher (Fin) and across the nursery and finisher (All) for six challenge loads based on clinical disease traits or growth rate

Trait	Slope genetic variance (SE) using clinical disease traits			Slope genetic variance (SE) using growth rate		
	NurCLc	FinCLc	CLc	NurCLg	FinCLg	CLg
Linear RN						
cNurADG	400 (300)	700 (300)	800 (300)	20 (300)	*2300 (300)*	2200 (400)
FinADG	*4000 (700)*	1000 (400)	800 (400)	2000 (500)	0	400 (400)
cNurTRT	0	0.10 (0.03)	0.09 (0.03)	NA	*0.15 (0.04)*	0.13 (0.04)
AllTRT	0.10 (0.05)	0.16 (0.05)	0.21 (0.06)	0.14 (0.05)	0.14 (0.05)	*0.31 (0.07)*
Cubic RN						
cNurADG	700 (400)	900 (300)	1000 (300)	300 (300)	*3000 (600)*	2000 (400)
FinADG	*4000 (600)*	1000 (400)	1000 (400)	3000 (600)	800 (400)	1000 (400)
cNurTRT	0	0.11 (0.04)	0.11 (0.04)	0.024 (0.03)	*0.15 (0.04)*	0.13 (0.04)
AllTRT	0.10 (0.05)	0.19 (0.05)	0.26 (0.06)	0.15 (0.06)	0.14 (0.05)	*0.31 (0.07)*

All six different CL were fitted as fixed effects in the linear and cubic reaction norm model, but only the corresponding CL was fitted as random animal genetic slope. cNurADG: growth rate in the challenge nursery; FinADG: growth rate in the finisher; cNurTRT: medical treatment rate in the challenge nursery; AllTRT: medical treatment rate across the challenge nursery and finisher; NurCLc: challenge load derived from the clinical disease traits in the challenge nursery; FinCLc: challenge load derived from the clinical disease traits in early finisher; CLc: weighted challenge load of NurCLc and FinCLc; NurCLg: challenge load derived from the growth rate in the challenge nursery; FinCLg: challenge load derived from the growth rate in early finisher; CLg: weighted challenge load of NurCLg and FinCLg; figures in italic characters indicate the greatest genetic slope variance for that trait

Table 3 shows the comparison of estimates of the slope genetic variance from the linear and cubic spline reaction norm models when fitting one CL as a fixed covariate and that same CL as the random genetic reaction norm. For the cubic spline model, only the slope variance was compared and the spline variance was not included in the comparison. Estimates of the slope genetic variance were slightly higher in the cubic spline model than in the linear model, as also observed in Table 2. In general, the linear and cubic spline reaction norm models showed the same pattern in estimates of slope genetic variance and, for a given trait, the same CL showed the largest estimate for both models. Consistent with results in Table 2, FinCLg was the best CL for analysis of cNurADG. However, different from the results in Table 2, CLc had the largest estimate of the slope genetic variance for analysis of FinADG, cNurTRT, and AllTRT for both the linear and cubic spline models, except for cNurTRT for the cubic spline model. For cNurTRT, NurCLc had the largest estimate of the slope genetic variance. The linear reaction norm model did not converge for analysis of cNurTRT based on NurCL.

**Table 3:** Comparison of slope genetic variance and reaction norm models for analysis of average daily gain (ADG, g) and treatment rate (TRT) in the challenge nursery (cNur), finisher (Fin) and across the nursery and finisher (All) for six challenge loads based on clinical disease traits or growth rate

Trait	CL using clinical disease traits			CL using growth rate			P-value vs. no RN
	NurCLc	FinCLc	CLc	NurCLg	FinCLg	CLg	
Linear RN							
cNurADG	400 (500)	500 (300)	500 (300)	0	*2200 (400)*	2000 (4000)	< 0.0001
FinADG	1100 (600)	200 (300)	*1200 (500)*	200 (400)	0	0	0.004
cNurTRT	NA	0.01 (0.02)	*0.10 (0.04)*	0	0.07 (0.03)	0.09 (0.03)	0.03
AllTRT	0	0	*0.21 (0.05)*	0.016 (0.03)	0	0.11 (0.04)	0.001

Trait	CL using clinical disease traits			CL using growth rate			P-value vs. linear RN
	NurCLc	FinCLc	CLc	NurCLg	FinCLg	CLg	
Cubic RN							
cNurADG	400 (500)	240 (300)	600 (300)	160 (300)	*2500 (600)*	2000 (500)	0.35
FinADG	1200 (700)	200 (200)	*1600 (500)*	500 (400)	0	0	0.001
cNurTRT	*0.24 (0.05)*	NA	0.08 (0.04)	0	0.07 (0.03)	0.09 (0.03)	0.04
AllTRT	0	0	*0.27 (0.06)*	0.015 (0.03)	0	0.11 (0.04)	0.0003

Only one corresponding CL was fitted as fixed effect in the linear and cubic spline reaction norm model. CL: challenge load; RN: reaction norm; NurCLc: challenge load derived from the clinical disease traits in the challenge nursery; FinCLc: challenge load derived from the clinical disease traits in early finisher; CLc: weighted challenge load of NurCLc and FinCLc; NurCLg: challenge load derived from the growth rate in the challenge nursery; FinCLg: challenge load derived from the growth rate in early finisher; CLg: weighted challenge load of NurCLg and FinCLg; P-value for cNurADG was based on FinCLg and P-values for FinADG, cNurTRT, and AllTRT were based on CLc because CLc was the best CL for these three traits. P-values for the linear model were based on comparison of the linear reaction norm model with the standard model without random slope term. P-values for the cubic spline model were based on comparison of the cubic spline reaction norm model with the linear reaction norm model. cNurADG: growth rate in the challenge nursery; FinADG: growth rate in the finisher; cNurTRT: medical treatment rate in the challenge nursery; AllTRT: medical treatment rate across the challenge nursery and finisher; figures in italic characters indicate the greatest genetic slope variance for that trait

In the remainder, based on having the largest estimate of the slope genetic variance in Table 3, only results for reaction norms on FinCLg will be shown for analysis of cNurADG and only those based on CLc for analysis of FinADG, cNurTRT, and AllTRT. Results of reaction norm analyses based on the other five CL are in Additional file 1: Tables S1–S5, with most resulting in either convergence issues or poor fit of the data.

### Comparison of alternate statistical models to capture the effect of CL

Likelihood ratio test results for the comparison of the linear reaction norm model to the standard model and of the cubic spline model to the linear reaction norm model are in Table 3. The linear reaction norm model fitted the data significantly better than the standard model for all four traits (P < 0.03) and the cubic spline reaction norm model fitted the data significantly better than the linear reaction norm model for FinADG, cNurTRT, and AllTRT (P < 0.04). Although the cubic spline model did not perform significantly better than the linear model for cNurADG (P = 0.35), it was significantly better than the standard model (P < 0.0001).

Comparisons of the reaction norm models to the multi-trait model based on AIC are in Table 4. For both cNurADG and AllTRT, the cubic spline model had the lowest AIC, followed by the linear model, then the multi-variate model. The linear reaction norm model had the lowest AIC for FinADG, followed by the cubic spline model, then the multi-variate model. In contrast, the multi-variate model had the lowest AIC for cNurTRT, followed by the cubic spline model, then the linear reaction norm model. These comparisons agreed with those of the likelihood ratio tests (Table 3), except for FinADG, for which the cubic spline model had a better fit than the linear model based on the likelihood ratio test.

**Table 4:** Estimates of heritability and genetic correlations from the multi-variate (MTV, 3-trait) and the linear and cubic spline reaction norm model analyses for low, medium, and high challenge loads, and the Akaike Information Criterion (AIC) for each model and trait

	cNurADG			FinADG			cNurTRT			AllTRT		
	MTV	Linear RN	Cubic RN	MTV	Linear RN	Cubic RN	MTV	Linear RN	Cubic RN	MTV	Linear RN	Cubic RN
Heritability												
Low^a^	0.33	0.35	0.44	0.55	0.38	0.42	0.18	0.18	0.20	0.28	0.24	0.33
Medium^b^	0.17	0.22	0.38	0.28	0.26	0.54	0.22	0.14	0.34	0.46	0.33	0.48
High^c^	0.23	0.42	0.56	0.42	0.25	0.70	0.26	0.22	0.42	0.52	0.53	0.79
Genetic correlation												
Low-medium	0.74	0.82	0.79	0.03	0.95	0.73	0.84	0.86	0.66	0.84	0.89	0.69
Low–high	0.79	0.08	0.14	− 0.09	0.40	0.06	0.03	0.16	0.17	− 0.10	0.27	0.18
Medium–high	0.59	0.63	0.69	0.67	0.67	0.60	− 0.24	0.63	0.81	− 0.02	0.68	0.80
AIC	− 9401.05	− 9462.96	*− 9467.15*	− 7520.05	*− 7615.61*	− 7542.87	*3482.47*	3526.30	3514.87	4430.96	4308.34	*4303.65*

cNurADG: growth rate in the challenge nursery; FinADG: growth rate in the finisher; cNurTRT: medical treatment rate in the challenge nursery; AllTRT: medical treatment rate across the challenge nursery and finisher; AIC: Akaike information criterion. cNurADG was analyzed based on CL derived from early finisher growth rate (FinCLg); FinADG, cNurTRT, and AllTRT were based on CL derived from the clinical disease traits across the challenge nursery and finisher (CLc); figures in italic characters indicate the lowest AIC value for that trait

^a^Low challenge load

^b^Medium challenge load

^c^High challenge load

### Heritability estimates

**Fig. 4:**
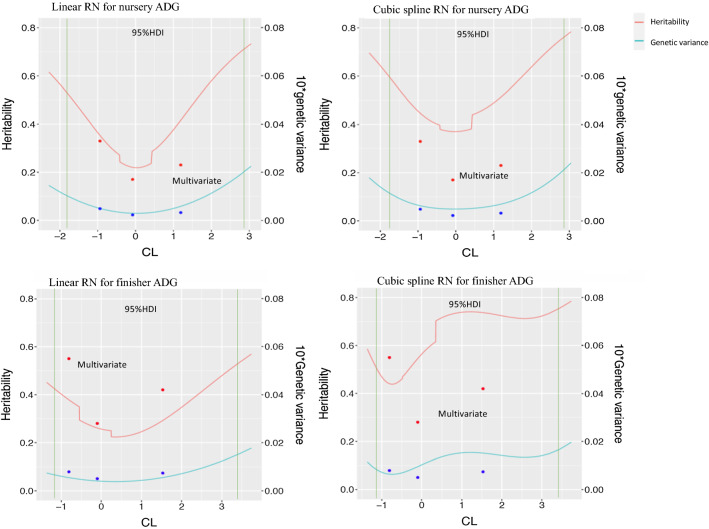
Estimates of heritability and genetic variance as a function of challenge load (CL) based on the linear and cubic spline reaction norm (RN) models for average daily gain (ADG, kg/day) in the challenge nursery and finisher. Dots indicate estimates from multi-variate analyses with phenotypes under low, intermediate, and high CL treated as different traits. Green vertical lines indicate the 95% highest density interval for the challenge load. Nursery ADG was analyzed based on CL derived from early finisher growth rate, while finisher ADG was based on CL derived from the clinical disease traits across the challenge nursery and finisher

**Fig. 5:**
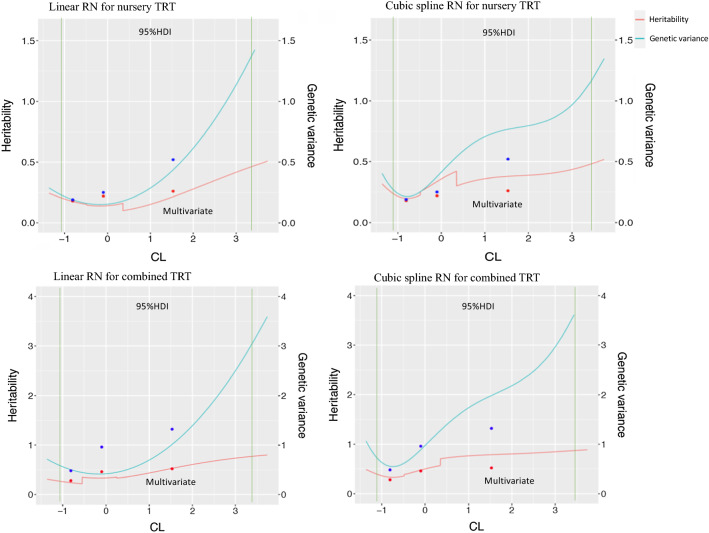
Estimates of heritability and genetic variance as a function of challenge load (CL) based on the linear and cubic spline reaction norm (RN) models for treatment rate in the challenge nursery and across challenge nursery and finisher. Dots indicate estimates from multi-variate analysis with phenotypes under low, intermediate, and high CL treated as different traits. Green vertical lines indicate the 95% highest density interval for the CL. TRT: medical treatment rate; combined TRT: medical treatment rate across the challenge nursery and finisher; both nursery and combined TRT were analyzed based on CL derived from the clinical disease traits across the challenge nursery and finisher

Estimates of heritability for ADG and TRT from the linear and cubic spline reaction norm models and from the multi-variate model are shown in Figs. 4 and 5. Estimates of heritability for cNurADG from the cubic spline reaction norm model were approximately twice as large as those from the linear model because of a larger estimate of genetic variance but the two models resulted in the same U-shaped curve of estimates as a function of CL. It should be noted that the cubic spline model did not fit the data significantly better than the linear model for cNurADG (Table 3). For FinADG, in general, estimates of heritability from the cubic spline model were about twice as large as those from the linear model for high CL and 67% larger for the intermediate CL class due to larger estimates of genetic variance. The two models, however, resulted in similar U-shaped curves for estimates of heritability as a function of CL. For TRT, in general, estimates of heritability from the linear and cubic spline models were similar and had similar trends for both cNurTRT and AllTRT, with increasing estimates as CL increased.

When comparing the reaction norm models with the multi-variate model, estimates of heritability from the multi-variate model for cNurADG in general had a similar trend as those from the linear and cubic spline reaction norm models, with the estimate of heritability being lower for intermediate CL than for low or high CL (Fig. 4; Table 4). Table 4 shows that estimates of heritability for cNurADG were moderate to high for all three models when CL was low, lowest when CL was intermediate, and moderate to high again when CL was high. However, the multi-variate model resulted in lower estimates of heritability for cNurADG than the reaction norm models, especially compared to the cubic spline model, mainly as a result of lower estimates of genetic variances. Estimates of heritability for FinADG from the multi-variate model did not match those from the cubic spline model very well, mainly because the estimates of genetic variance for the intermediate and high CL were very high for the cubic spline model. Estimates of heritability for FinADG from the multi-variate model were larger than those of the linear model but lower than those of the cubic spline model, but they did follow a similar U-shaped pattern as those from the reaction norm models. For TRT, in general, estimates of heritability from the multi-variate model showed similar trends as those from both the linear and cubic spline models for both cNurTRT and AllTRT, with increasing estimates with increasing CL. Table 4 shows that estimates of heritability for cNurTRT at low, intermediate, and high CL were lower than for AllTRT for all three models. Estimates of heritability for TRT from the multi-variate model were lower than those from the cubic spline model for high CL because of a lower estimate of genetic variance, but similar for low and intermediate CL.

### Genetic correlations of traits between different levels of CL

**Fig. 6:**
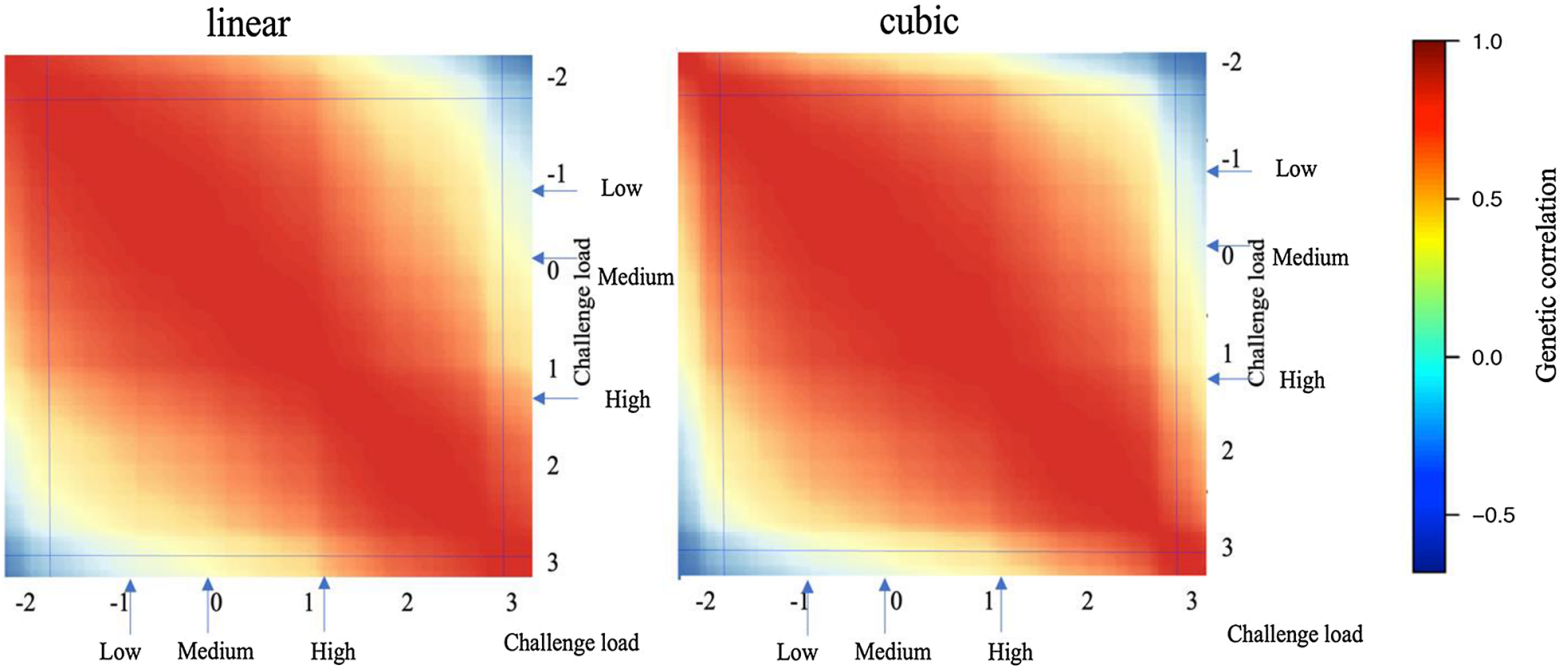
Estimates of genetic correlations from the linear and the cubic spline reaction norm model for average daily gain (ADG, kg/day) in the challenge nursery using challenge load derived from early finisher growth rate. Horizontal and vertical lines on the heatmaps indicate 95% highest density interval for the challenge load; Arrows on the heatmap scales indicate the mean challenge load for the low, medium, and high categories used for the multi-trait analyses

**Fig. 7:**
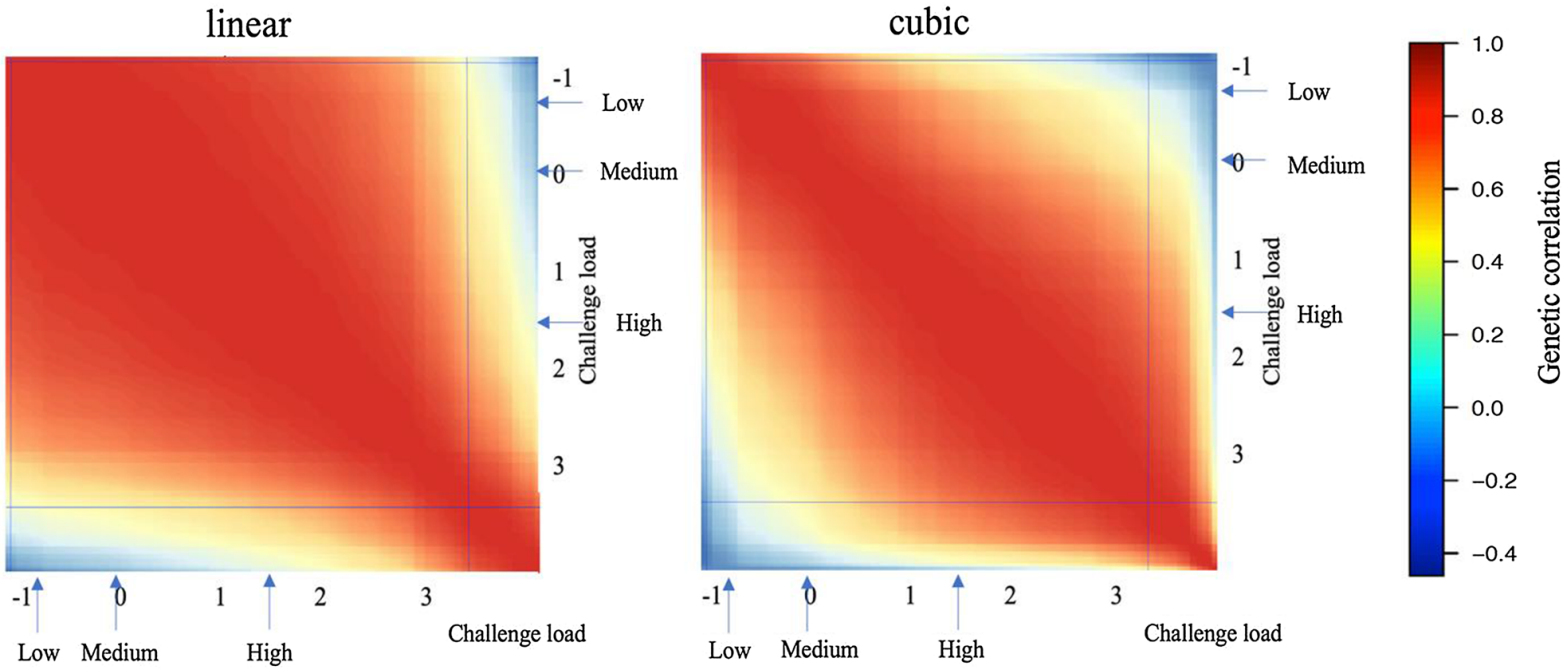
Estimates of genetic correlations from the linear and the cubic spline reaction norm model for average daily gain (ADG, kg/day) in the finisher using challenge load derived from the clinical disease traits across the challenge nursery and finisher. Horizontal and vertical lines on the heatmaps indicate 95% highest density interval for the challenge load; Arrows on the heatmap scales indicate the mean challenge load for the low, medium, and high categories used for the multi-trait analyses

Estimates of genetic correlations from the linear and cubic spline reaction norm models for ADG are shown in Figs. 6 and 7 and Additional file 2: Figs. S1 and S2. For cNurADG, estimates of genetic correlations were similar for the linear and cubic spline models, consistent with the finding that the cubic spline model did not fit the data significantly better than the linear reaction norm model (Table 3). Estimates of genetic correlations were high for pairs of CL that were close to each other and low and even negative for pairs of CL that were very different, e.g. low CL with high CL. For FinADG, the linear and cubic spline models showed different patterns for estimates of genetic correlations, with higher estimates for the cubic spline model than for the linear model for pairs of CL that were close.

**Fig. 8:**
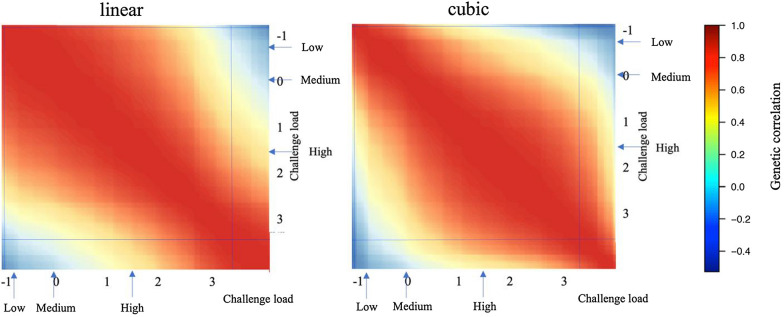
Estimates of genetic correlations from the linear and the cubic spline reaction norm model for treatment rate in the challenge nursery using challenge load derived from the clinical disease traits across the challenge nursery and finisher. Horizontal and vertical lines on the heatmaps indicate 95% highest density interval for the challenge load; Arrows on the heatmap scales indicate the mean challenge load for the low, medium, and high categories used for the multi-trait analyses

**Fig. 9:**
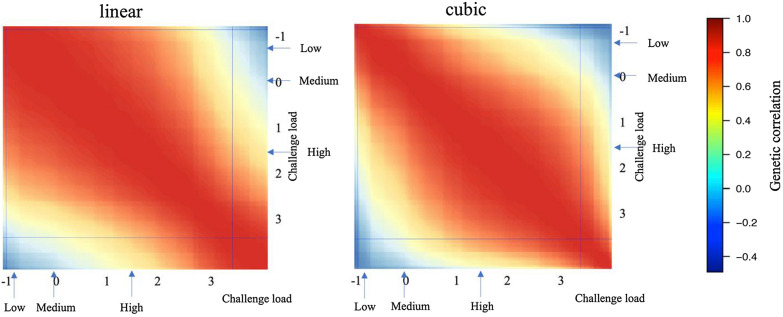
Estimates of genetic correlations from the linear and the cubic spline reaction norm model for treatment rate across the challenge nursery and finisher using challenge load derived from the clinical disease traits across the challenge nursery and finisher. Horizontal and vertical lines on the heatmaps indicate 95% highest density interval for the challenge load; Arrows on the heatmap scales indicate the mean challenge load for the low, medium, and high categories used for the multi-trait analyses

Estimates of genetic correlations from the linear and cubic spline reaction norm models for TRT are shown in Figs. 8 and 9 and Additional file 2: Figs. S3 and S4. Similar to FinADG, the linear and cubic spline reaction norm models resulted in different patterns of genetic correlation estimates for both cNurTRT and AllTRT. For pairs of CL that were close, estimates of genetic correlations from the cubic spline model were always stronger than those from the linear reaction norm model.

Point estimates of genetic correlations from the multi-variate and the linear and cubic spline reaction norm models for low, intermediate, and high CL are in Table 4. Linear and cubic spline models showed a very similar pattern in genetic correlation estimates for most traits, in that the estimate between low and high CL was much lower than the estimates of intermediate with either low or high CL. This was, however, not true for the multi-variate analysis, for which all genetic correlation estimates were high for cNurADG, while for FinADG, the estimate was high between intermediate and high CL, low between low and intermediate CL, and very low between low and high CL. Both cNurTRT and AllTRT had high estimates of the genetic correlation between the low and intermediate CL for the multi-variate model but low estimates between low and high CL and between intermediate and high CL.

## Discussion

### Quantification of disease challenge loads

Several previous studies have quantitatively derived disease CL or environment using performance records in livestock [2, 14, 15, 17, 25, 26]. Mathur et al. [17] developed a method to estimate challenge loads due to PRRS outbreaks using reproduction records from sow farms based on the assumption that a reduction in reproductive output of sows was proportional to the magnitude of the challenge load during an outbreak. The same method to derive the challenge load was used by Herrero-Medrano et al. [25] to estimate genetic parameters and breeding values across PRRS challenged environments. Unlike Mathur et al. [17], Rashidi et al. [14] used standardized herd-year-week estimates of number born alive as environmental covariates for a reaction norm analysis and found genetic variation among sows in response to PRRS outbreaks. Guy et al. [15] characterized disease challenge load by environmental descriptors based on contemporary group estimates for growth rate, feed intake, backfat, or muscle depth, and found that the ability to detect the genotype-by-environment interactions for growth was greatest when using contemporary group estimates for backfat and feed intake.

Very few studies have used clinical disease phenotypes to derive CL for disease resilience analyses [16, 20] and these studies were either disease specific (mastitis) [20] or were not intentionally applying a disease challenge [16], resulting in a small range of CL. Guy et al. [16] also used medication records to quantify the disease challenge load. In our study, challenge loads in a severe polymicrobial disease challenge were quantified based on growth rate and clinical disease traits, including medical treatment and mortality rates, and subjective health scores. These clinical measures were a direct reflection of the disease challenge and clearly showed that some batches were more heavily challenged than others (Fig. 2). Given the severity of the challenge, the results from this study may not apply to a situation with lower CL.

The choice of the environmental variable in reaction norm analyses, in this case disease CL, can affect the power to detect variation in reaction norm slopes [15, 27]. In the present study, six disease CL were derived from phenotypes on growth rate and clinical disease traits recorded in the challenge nursery and finisher and were compared based on model loglikelihoods and estimates of genetic slope variance. Indeed, different CL resulted in different loglikelihoods and estimates of genetic slope variances for the same analyzed trait for both the linear and the cubic spline reaction norm models (Tables 1, 2). It should be noted that CLc and CLg were highly correlated with FinCLc (0.99) and FinCLg (0.90) (Fig. 3), resulting in these pairs of CL having similar loglikelihoods and slope variance estimates. NurCLc and FinCLc were also moderately correlated with NurCLg (0.75) and FinCLg (0.71) (Fig. 3). Comparisons of different measures of CL based on loglikelihood or based on estimates slope genetic variance in general agreed with each other. The CL that had the highest loglikelihood and estimate of slope genetic variance was, however, not consistent across traits. In addition, the same CL was the best CL for a given phase across traits, e.g. FinCLg was the best CL for both cNurADG and cNurTRT (Table 2).

For the results in Table 2, all six CL were fitted as covariates in the model to enable comparison of the loglikelihoods. However, in practice, only one CL is fitted as a fixed covariate and as a random effect for reaction norm analyses. For such models, Table 3 shows that CLc was the best CL for all traits, except for cNurADG. However, although FinCLg was the best CL for cNurADG, FinCLg was highly correlated with CLc (Fig. 3, 0.70). For cNurTRT, although NurCLc was the best CL for the cubic spline model, NurCLc had convergence issues for the linear model for cNurTRT. Use of one of the other five CL as the environmental covariate for reaction norm analyses either resulted in convergence issues or in a poor fit based on the p-value from the likelihood ratio tests (see Additional file 1: Tables S1–S5). Therefore, the CL derived from the clinical disease traits across the challenge nursery and finisher was considered the best CL for most traits. Biologically, this makes sense because the clinical disease traits are a more direct reflection of the disease challenge than other performance traits. In addition, basing the CL on clinical data across the nursery and finisher may provide more information to capture the severity of the disease challenge.

### Comparison of reaction norm models

Based on the loglikelihood ratio test, the linear reaction norm model fitted the data better than the standard model without a random genetic slope term, for all traits (Table 3). This shows the presence of genotype-by-environment interactions, i.e. that a pig’s genetic value for ADG or TRT depends on the level of CL that it is exposed to. Based on the loglikelihood ratio test, the cubic spline reaction norm model fitted the data better than the linear reaction norm model for FinADG, cNurTRT, and AllTRT (Table 3), suggesting a non-linear relationship between CL and breeding values for these traits. Carvalheiro et al. [26] also found that the spline model outperformed the linear reaction norm model for post-weaning weight gain in relation to the quality of the environment in beef cattle, which was based on estimates of the contemporary group effect for post-weaning weight gain. They also found that a quadratic reaction norm model provided a better fit than the linear reaction norm model. The quadratic reaction norm model, however, had convergence issues for most traits in our data and was, therefore, not pursued further.

For comparison, the data were also analyzed with a three-trait multi-variate model, with traits defined based on CL class (low, medium, or high), which does not assume a specific relationship between CL and phenotype. Based on AIC, the multi-variate model was found to provide a poorer fit than the linear and cubic spline reaction norm models for all traits, except for cNurTRT (Table 4), for which the multi-variate model was best. The reason for the latter is not clear. The superiority of the reaction norm model over the multi-variate model is consistent with results by Rashidi et al. [14], who found that the reaction norm model fitted the data better than a bivariate model when analyzing reproductive performance of sows following PRRS infection.

### Reaction norm genetic parameters

#### Heritability as a function of CL

In general, estimates of heritability from the linear reaction norm model matched estimates from the multi-variate model, with both models resulting in a U-shaped pattern of estimates of heritability for ADG as a function of CL and increasing estimates of heritability with increasing CL for TRT. The cubic spline reaction norm model tended to give higher estimates of heritability than the linear reaction norm model for all traits but resulted in a similar pattern of estimates as the latter. The U-shaped trend for estimates of heritability for ADG is consistent with findings of Herrero-Medrano et al. [25] for number born alive for sows based on PRRS challenge load. Estimates of heritability for cNurTRT and AllTRT generally increased with CL, which was consistent with the findings of Herrero-Medrano et al. [25] for number of pigs lost as a function of PRRS challenge load.

#### Genetic correlations of traits under different CL

The linear and cubic spline reaction norm models generally estimated a similar pattern of genetic correlations for a trait between different levels of CL; high when the CL were close and low when the CL were very different (Figs. 6, 7, 8, 9). However, for most traits, the cubic spline model generated stronger estimates of genetic correlations for pairs of CL that were close. The better fit of the cubic spline model for most traits (Table 3) suggests that the linear reaction norm model underestimated genetic correlations for pairs of CL that were close. This was as expected, as the cubic spline model is more flexible and less driven by the assumption of linearity. Similar trends were observed for non-disease challenge studies that used reaction norm models [28, 29]. The estimates of genetic correlations for a trait between different CL are, however, also biologically reasonable because phenotypes are expected to be more correlated when recorded under similar levels of CL. Low genetic correlations among environments with different levels of CL suggests that selection on estimates of breeding value based on phenotypes obtained in a given CL would result in low response to selection for performance under a very different CL.

**Fig. 10:**
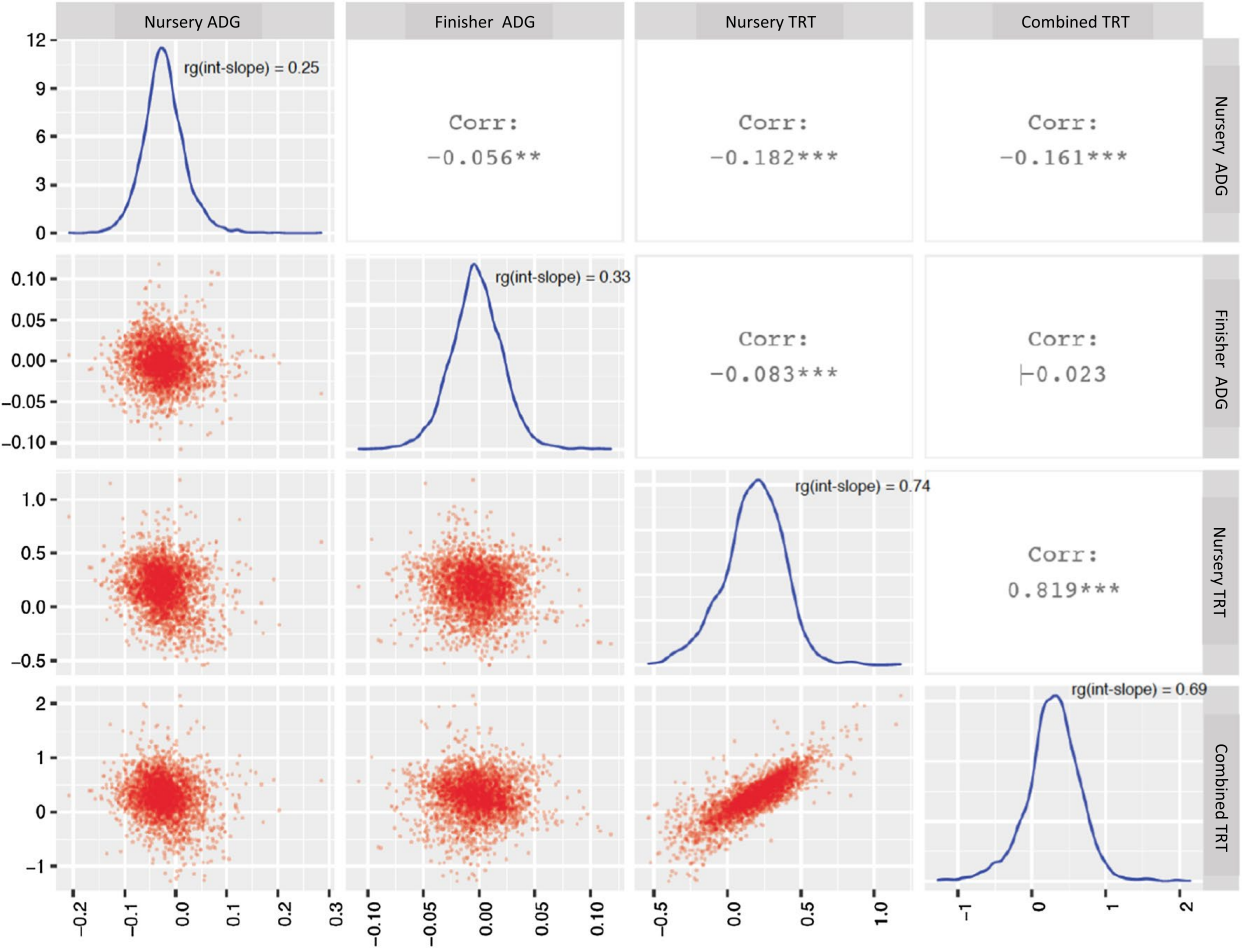
Distribution and relationships of estimated breeding values for slope from the linear reaction norm model for average daily gain (ADG, kg/day) and treatment rate (TRT) in or across (combined) the challenge nursery and finisher. rg(int-slope) on the diagonal refers to the estimate of the genetic correlation between the intercept and slope for that trait

Estimates of genetic correlations between the intercept (CL = 0) and slope based on the linear reaction norm model are shown in Fig. 10 for ADG and TRT, noting that CL = 0 corresponds to intermediate CL, rather than no disease challenge. Low genetic correlation estimates between intercept and slope were observed for ADG (0.25–0.33), while estimates for TRT were high and positive (0.69–0.74). A low genetic correlation between the intercept and slope for ADG suggests that growth rate at a given CL (CL = 0) and resilience are genetically different traits, suggesting an opportunity for joint selection for resilience and increased growth performance at a given CL. High positive estimates of the genetic correlation between the intercept and slope for TRT implies that resilience and treatment rate at a given CL (CL = 0) are very similar traits and suggest that a lower treatment rate when CL = 0 is genetically associated with a lower slope (higher resilience). This is favorable when selecting for resilience, as selection can focus on treatment rate under intermediate CL (CL = 0), regardless of the slope, which is usually difficult to obtain. Here, performance at CL = 0 was used as the intercept but this can be changed to any level of CL, which will generate different genetic correlations between intercept and slope but equivalent estimated breeding values (EBV) at a given CL.

### Estimated breeding values as a function of CL

**Fig. 11:**
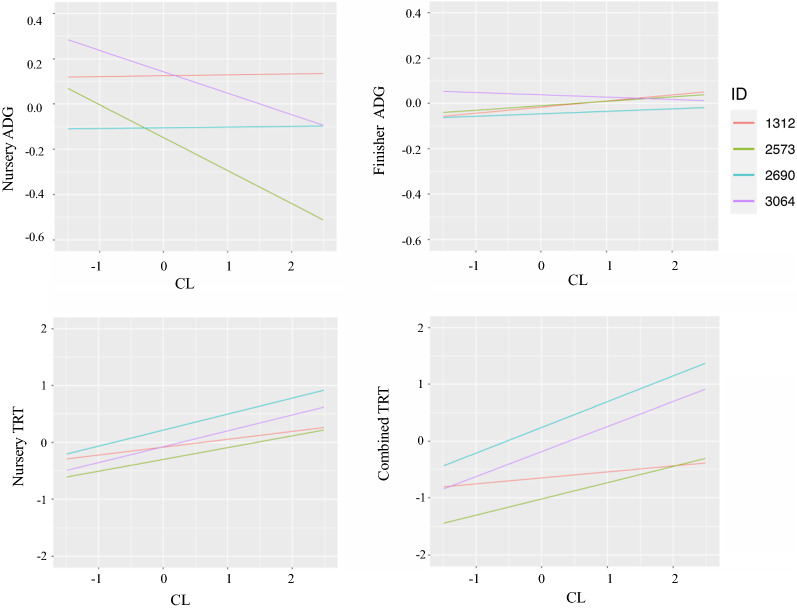
Estimates of breeding values for four animals as a function of challenge load from the linear reaction norm model for average daily gain (ADG, kg/day) and treatment rate (TRT) in or across (combined) the challenge nursery and finisher. Challenge load was derived using clinical disease traits across the challenge nursery and finisher

Relationships between EBV for slope for different traits from the linear reaction norm model are shown in Fig. 10. A high correlation (0.82) between the EBV of slopes for cNurTRT and AllTRT was observed, similar to the slope and spline coefficient of the cubic spline reaction norm model (see Additional file 2: Figs. S5, S6). The EBV for cNurADG as a function of CL for four representative animals based on the linear reaction norm model are shown in Fig. 11, representing an animal with high performance at CL = 0 and high resilience (animal 1312), an animal with high performance at CL = 0 and low resilience (animal 3064), an animal with low performance at CL = 0 and high resilience (animal 2690), and an animal with low performance at CL = 0 and low resilience (animal 2573). The EBV at a given CL was computed as the EBV for the intercept plus CL times the estimate of the slope, which was the sum of the fixed effect estimate and the EBV of the slope. The EBV for FinADG, cNurTRT, and AllTRT for the same four animals are shown in Fig. 11. It should be noted that a resilient animal has a less negative slope for ADG and a less positive slope for TRT. Animal 3064 was not resilient based on cNurADG, FinADG, and TRT. Animal 2573 was not resilient based on cNurADG and TRT but resilient based on FinADG. Animal 1312 was resilient based on ADG but less resilient based on TRT. Animal 2690 was also resilient based on ADG but less resilient based on TRT. As expected, EBV for cNurTRT had very similar patterns as EBV for AllTRT, indicating pigs that were genetically resilient or that had a high treatment rate at a given CL based on cNurTRT were also resilient based on AllTRT. This is because the slope EBV for cNurTRT were highly correlated with those for AllTRT (corr = 0.82). The EBV based on the cubic spline model for the same four animals are shown in Additional file 2: Fig. S7. The EBV were computed as the sum of estimates for intercept, CL*slope, and z*spline coefficient. The EBV based on the cubic spline model had a similar pattern to those based on the linear model but in general had less variation, e.g. none of the animals were resilient based on AllTRT but their curves were almost identical.

### Implementation in breeding programs

Using EBV derived from reaction norm models, different selection strategies can be implemented, depending on the breeding goal. If the breeding goal is to select pigs that are more resilient to disease across different CL, selection should be for low EBV for slope of ADG or TRT only, regardless of the intercept (CL = 0). Another strategy is to select animals with both higher performance and higher resilience by selecting on EBV for both intercept and slope. A special case is to select pigs that perform better at a given CL level, in which case selection should be on the sum of EBV for intercept and for CL*slope at a given CL, provided the CL is well defined. The low positive estimates of the genetic correlation between the intercept and slope of the linear reaction norm model for growth rate (0.25 for cNurADG; 0.33 for FinADG) suggest that it is possible to select for both increased performance and resilience. Typically, phenotypes for ADG are available on selection candidates in the nucleus farm, representing very low CL. Thus, EBV for ADG in the nucleus can be added to the selection index, coupled with EBV obtained for resilience, which will target genetic improvement of ADG in a commercial farm with a harsher environment. High positive estimates of the genetic correlation between intercept and slope for treatment rates (0.74 for cNurTRT and 0.69 for AllTRT) also suggests a correlated response of higher resilience with selection only on EBV for treatment rate at CL = 0. Selection programs of breeding organizations are typically conducted under favorable environments in the nucleus farm, i.e. low CL, which could result in low responses to selection in commercial farms that have unfavorable environmental conditions (high CL). One solution to this is to incorporate the trait collected at the commercial farm as a separate trait and conduct a two-trait analysis, estimating EBV for commercial performance for animals in the nucleus. Strategies to optimize selection in the nucleus on data collected in the nucleus and under a disease challenge to maximize response in a target environment were recently explored by Dekkers [30].

## Conclusions

Clinical disease traits such as health scores and health treatment, mortality, and growth rates can be used to estimate the disease challenge load that pigs in a pen are exposed to. The linear reaction norm models fitted the disease challenge data significantly better than the standard genetic model without a reaction norm. Reaction norm models also fitted the data better than multivariate models. Cubic spline reaction norm models fitted the data significantly better than the linear reaction norm model for most traits, indicating non-linear relationships between disease resilience traits and the estimated challenge load. With increasing challenge load, estimates of heritability for growth rate initially went down and then increased, while estimates of heritability for treatment rate generally increased with challenge load. Estimates of genetic correlations for a phenotype between extreme challenge loads were low and even negative, but high when the challenge loads were close. The results of this study identified important genotype-by-environment interactions for disease resilience traits and that reaction norm models can be implemented to select more resilient animals across different challenge load levels or high-performance animals at a given challenge load level, or both.

## Supplementary Information

**Additional file 1**: **Table S1.** Comparison of the linear and cubic spline reaction norm model using NurCLc. **Table S2.** Comparison of the linear and cubic spline reaction norm model using FinCLc. **Table S3.** Comparison of the linear and cubic spline reaction norm model using NurCLg. **Table S4.** Comparison of the linear and cubic spline reaction norm model using FinCLg. **Table S5.** Comparison of the linear and cubic spline reaction norm model using CLg

**Additional file 2**: **Figure S1.** Estimates of genetic correlations from the linear and the cubic spline reaction norm model for average daily gain (ADG, kg/d) in the challenge nursery using challenge load derived from early finisher growth rate. **Figure S2.** Estimates of genetic correlations from the linear and the cubic spline reaction norm model for average daily gain (ADG, kg/d) in the finisher using challenge load derived from the clinical disease traits across the challenge nursery and finisher. **Figure S3.** Estimates of genetic correlations from the linear and the cubic spline reaction norm model for treatment rate in the challenge nursery using challenge load derived from the clinical disease traits across the challenge nursery and finisher. **Figure S4.** Estimates of genetic correlations from the linear and the cubic spline reaction norm model for treatment rate across the challenge nursery and finisher using challenge load derived from the clinical disease traits across the challenge nursery and finisher. **Figure S5**. Distribution and relationships of estimated breeding values for slope (including fixed effect estimate) from the cubic spline reaction norm model for average daily gain (ADG, kg/d) and treatment rate (TRT) in or across (combined) the challenge nursery and finisher. **Figure S6.** Distribution and relationships of estimated breeding values for spline coefficient (including fixed effect estimate) from the cubic spline reaction norm model for average daily gain (ADG, kg/d) and treatment rate (TRT) in or across (combined) the challenge nursery and finisher. **Figure S7**. Estimates of breeding values for four animals as a function of challenge load from the cubic spline reaction norm model for average daily gain (ADG, kg/d) and treatment rate (TRT) in or across (combined) the challenge nursery and finisher.

## Data Availability

The data used are on commercially owned animals and are, therefore, not publicly available. Data are, however, available from the authors upon reasonable request.
