# Supplementary material for: Genetic analysis of disease resilience of wean-to-finish pigs under a natural disease challenge model using reaction norms
Source: Genet Sel Evol. 2022 Feb 8;54:11. doi: 10.1186/s12711-022-00702-0 (PMC8822643; doi:10.1186/s12711-022-00702-0)
Supplement: Supplementary file 1 — Additional file 1: Table S1. Comparison of the linear and cubic spline reaction norm model using NurCLc. Table S2. Comparison of the linear and cubic spline reaction norm model using FinCLc. Table S3. Comparison of the linear and cubic spline reaction norm model using NurCLg. Table S4. Comparison of the linear and cubic spline reaction norm model using FinCLg. Table S5. Comparison of the linear and cubic spline reaction norm model using CLg [file 12711_2022_702_MOESM1_ESM.docx]

**Additional tables**

**Table S1. Comparison of the linear and cubic spline reaction norm model using NurCLc**

| Model |  | loglikelihood |  | No. model  parameters |  | 2*$\Delta\mathrm{loglik}e\mathrm{lihood}$ |  | p-value |
| --- | --- | --- | --- | --- | --- | --- | --- | --- |
| **Standard model** |  |  |  |  |  |  |  |  |
| cNurADG |  | 5145.92 |  | 5 |  | - |  | - |
| FinADG |  | 4010.13 |  | 5 |  | - |  | - |
| cNurTRT |  | -1882.66 |  | 5 |  | - |  | - |
| AllTRT |  | -1842.10 |  | 5 |  | - |  | - |
|  |  |  |  |  |  |  |  |  |
| **Linear RN** |  |  |  |  |  |  |  |  |
| cNurADG |  | 5150.13 |  | 7 |  | 8.42 |  | 0.01 |
| FinADG |  | 4010.93 |  | 7 |  | 1.60 |  | 0.45 |
| cNurTRT |  | NA |  | 7 |  | NA |  | NA |
| AllTRT |  | NA |  | 7 |  | NA |  | NA |
|  |  |  |  |  |  |  |  |  |
| **Cubic RN** |  |  |  |  |  |  |  |  |
| cNurADG |  | 5150.13 |  | 8 |  | 0 |  | 1 |
| FinADG |  | 4011.26 |  | 8 |  | 0.66 |  | 0.42 |
| cNurTRT |  | -1792.81 |  | 8 |  | NA |  | NA |
| AllTRT |  | NA |  | 8 |  | NA |  | NA |

P-values for the linear model were based on comparison of linear reaction norm model with standard model. P-values for the cubic spline model were based on comparison of cubic spline reaction norm model with the linear reaction norm model. NurCLc: challenge load derived from the clinical disease traits in the challenge nursery; cNurADG: growth rate in the challenge nursery; FinADG: growth rate in the finisher; cNurTRT: medical treatment rate in the challenge nursery; AllTRT: medical treatment rate across the challenge nursery and finisher

**Table S2. Comparison of the linear and cubic spline reaction norm model using FinCLc**

| Model |  | loglikelihood |  | No. model  parameters |  | 2*$\Delta\mathrm{loglik}e\mathrm{lihood}$ |  | p-value |
| --- | --- | --- | --- | --- | --- | --- | --- | --- |
| **Standard model** |  |  |  |  |  |  |  |  |
| cNurADG |  | 4725.36 |  | 5 |  | - |  | - |
| FinADG |  | 4016.19 |  | 5 |  | - |  | - |
| cNurTRT |  | -1528.59 |  | 5 |  | - |  | - |
| AllTRT |  | -1845.06 |  | 5 |  | - |  | - |
|  |  |  |  |  |  |  |  |  |
| **Linear RN** |  |  |  |  |  |  |  |  |
| cNurADG |  | 4727.51 |  | 7 |  | 4.30 |  | 0.12 |
| FinADG |  | 4016.52 |  | 7 |  | 0.66 |  | 0.72 |
| cNurTRT |  | -1527.75 |  | 7 |  | 1.68 |  | 0.43 |
| AllTRT |  | NA |  | 7 |  | NA |  | NA |
|  |  |  |  |  |  |  |  |  |
| **Cubic RN** |  |  |  |  |  |  |  |  |
| cNurADG |  | 4729.01 |  | 8 |  | 3.00 |  | 0.57 |
| FinADG |  | 4016.52 |  | 8 |  | 0 |  | 1 |
| cNurTRT |  | -1527.69 |  | 8 |  | 0.12 |  | 0.73 |
| AllTRT |  | NA |  | 8 |  | NA |  | NA |

P-values for the linear model were based on comparison of linear reaction norm model with standard model. P-values for the cubic spline model were based on comparison of cubic spline reaction norm model with the linear reaction norm model. FinCLc: challenge load derived from the clinical disease traits in the finisher; cNurADG: growth rate in the challenge nursery; FinADG: growth rate in the finisher; cNurTRT: medical treatment rate in the challenge nursery; AllTRT: medical treatment rate across the challenge nursery and finisher

**Table S3. Comparison of the linear and cubic spline reaction norm model using NurCLg**

| Model |  | loglikelihood |  | No. model  parameters |  | 2*$\Delta\mathrm{loglik}e\mathrm{lihood}$ |  | p-value |
| --- | --- | --- | --- | --- | --- | --- | --- | --- |
| **Standard model** |  |  |  |  |  |  |  |  |
| cNurADG |  | 5151.69 |  | 5 |  | - |  | - |
| FinADG |  | 4005.19 |  | 5 |  | - |  | - |
| cNurTRT |  | -1898.43 |  | 5 |  | - |  | - |
| AllTRT |  | -1847.46 |  | 5 |  | - |  | - |
|  |  |  |  |  |  |  |  |  |
| **Linear RN** |  |  |  |  |  |  |  |  |
| cNurADG |  | NA |  | 7 |  | NA |  | NA |
| FinADG |  | 4006.19 |  | 7 |  | 2 |  | 0.37 |
| cNurTRT |  | NA |  | 7 |  | NA |  | NA |
| AllTRT |  | -1847.38 |  | 7 |  | 0.16 |  | 0.21 |
|  |  |  |  |  |  |  |  |  |
| **Cubic RN** |  |  |  |  |  |  |  |  |
| cNurADG |  | 5159.98 |  | 8 |  | NA |  | NA |
| FinADG |  | 4006.97 |  | 8 |  | 1.56 |  | 0.21 |
| cNurTRT |  | NA |  | 8 |  | NA |  | NA |
| AllTRT |  | NA |  | 8 |  | NA |  | NA |

P-values for the linear model were based on comparison of linear reaction norm model with standard model. P-values for the cubic spline model were based on comparison of cubic spline reaction norm model with the linear reaction norm model. NurCLg: challenge load derived from the growth rate in the challenge nursery; cNurADG: growth rate in the challenge nursery; FinADG: growth rate in the finisher; cNurTRT: medical treatment rate in the challenge nursery; AllTRT: medical treatment rate across the challenge nursery and finisher

**Table S4. Comparison of the linear and cubic spline reaction norm model using FinCLg**

| Model |  | loglikelihood |  | No. model  parameters |  | 2*$\Delta\mathrm{loglik}e\mathrm{lihood}$ |  | p-value |
| --- | --- | --- | --- | --- | --- | --- | --- | --- |
| **Standard model** |  |  |  |  |  |  |  |  |
| cNurADG |  | 4706.65 |  | 5 |  | - |  | - |
| FinADG |  | 4037.46 |  | 5 |  | - |  | - |
| cNurTRT |  | -1568.50 |  | 5 |  | - |  | - |
| AllTRT |  | -1840.81 |  | 5 |  | - |  | - |
|  |  |  |  |  |  |  |  |  |
| **Linear RN** |  |  |  |  |  |  |  |  |
| cNurADG |  | 4738.48 |  | 7 |  | 63.66 |  | <0.0001 |
| FinADG |  | NA |  | 7 |  | NA |  | NA |
| cNurTRT |  | -1562.84 |  | 7 |  | 11.32 |  | 0.003 |
| AllTRT |  | NA |  | 7 |  | NA |  | NA |
|  |  |  |  |  |  |  |  |  |
| **Cubic RN** |  |  |  |  |  |  |  |  |
| cNurADG |  | 4739.52 |  | 8 |  | 2.08 |  | 0.35 |
| FinADG |  | 4036.49 |  | 8 |  | NA |  | NA |
| cNurTRT |  | -1562.84 |  | 8 |  | 0 |  | 1 |
| AllTRT |  | NA |  | 8 |  | NA |  | NA |

P-values for the linear model were based on comparison of linear reaction norm model with standard model. P-values for the cubic spline model were based on comparison of cubic spline reaction norm model with the linear reaction norm model. FinCLg: challenge load derived from the growth rate in the early finisher; cNurADG: growth rate in the challenge nursery; FinADG: growth rate in the finisher; cNurTRT: medical treatment rate in the challenge nursery; AllTRT: medical treatment rate across the challenge nursery and finisher

**Table S5. Comparison of the linear and cubic spline reaction norm model using CLg**

| Model |  | loglikelihood |  | No. model  parameters |  | 2*$\Delta\mathrm{loglik}e\mathrm{lihood}$ |  | p-value |
| --- | --- | --- | --- | --- | --- | --- | --- | --- |
| **Standard model** |  |  |  |  |  |  |  |  |
| cNurADG |  | 4710.40 |  | 5 |  | - |  | - |
| FinADG |  | 4029.62 |  | 5 |  | - |  | - |
| cNurTRT |  | -1565.79 |  | 5 |  | - |  | - |
| AllTRT |  | -1846.01 |  | 5 |  | - |  | - |
|  |  |  |  |  |  |  |  |  |
| **Linear RN** |  |  |  |  |  |  |  |  |
| cNurADG |  | 4731.54 |  | 7 |  | 42.28 |  | <0.0001 |
| FinADG |  | NA |  | 7 |  | NA |  | NA |
| cNurTRT |  | -1561.21 |  | 7 |  | 9.16 |  | 0.01 |
| AllTRT |  | -1842.40 |  | 7 |  | 7.22 |  | 0.03 |
|  |  |  |  |  |  |  |  |  |
| **Cubic RN** |  |  |  |  |  |  |  |  |
| cNurADG |  | 4731.81 |  | 8 |  | 0.54 |  | 0.46 |
| FinADG |  | NA |  | 8 |  | NA |  | NA |
| cNurTRT |  | -1561.21 |  | 8 |  | 0 |  | 1 |
| AllTRT |  | NA |  | 8 |  | NA |  | NA |

P-values for the linear model were based on comparison of linear reaction norm model with standard model. P-values for the cubic spline model were based on comparison of cubic spline reaction norm model with the linear reaction norm model. CLg: challenge load derived from the growth rate from both the challenge nursery and early finisher; cNurADG: growth rate in the challenge nursery; FinADG: growth rate in the finisher; cNurTRT: medical treatment rate in the challenge nursery; AllTRT: medical treatment rate across the challenge nursery and finisher
