# Supplementary material for: Genetic analysis of disease resilience of wean-to-finish pigs under a natural disease challenge model using reaction norms
Source: Genet Sel Evol. 2022 Feb 8;54:11. doi: 10.1186/s12711-022-00702-0 (PMC8822643; doi:10.1186/s12711-022-00702-0)
Supplement: Supplementary file 2 — Additional file 2: Figure S1. Estimates of genetic correlations from the linear and the cubic spline reaction norm model for average daily gain (ADG, kg/d) in the challenge nursery using challenge load derived from early finisher growth rate. Figure S2. Estimates of genetic correlations from the linear and the cubic spline reaction norm model for average daily gain (ADG, kg/d) in the finisher using challenge load derived from the clinical disease traits across the challenge nursery and finisher. Figure S3. Estimates of genetic correlations from the linear and the cubic spline reaction norm model for treatment rate in the challenge nursery using challenge load derived from the clinical disease traits across the challenge nursery and finisher. Figure S4. Estimates of genetic correlations from the linear and the cubic spline reaction norm model for treatment rate across the challenge nursery and finisher using challenge load derived from the clinical disease traits across the challenge nursery and finisher. Figure S5. Distribution and relationships of estimated breeding values for slope (including fixed effect estimate) from the cubic spline reaction norm model for average daily gain (ADG, kg/d) and treatment rate (TRT) in or across (combined) the challenge nursery and finisher. Figure S6. Distribution and relationships of estimated breeding values for spline coefficient (including fixed effect estimate) from the cubic spline reaction norm model for average daily gain (ADG, kg/d) and treatment rate (TRT) in or across (combined) the challenge nursery and finisher. Figure S7. Estimates of breeding values for four animals as a function of challenge load from the cubic spline reaction norm model for average daily gain (ADG, kg/d) and treatment rate (TRT) in or across (combined) the challenge nursery and finisher. [file 12711_2022_702_MOESM2_ESM.docx]

**Additional figures**


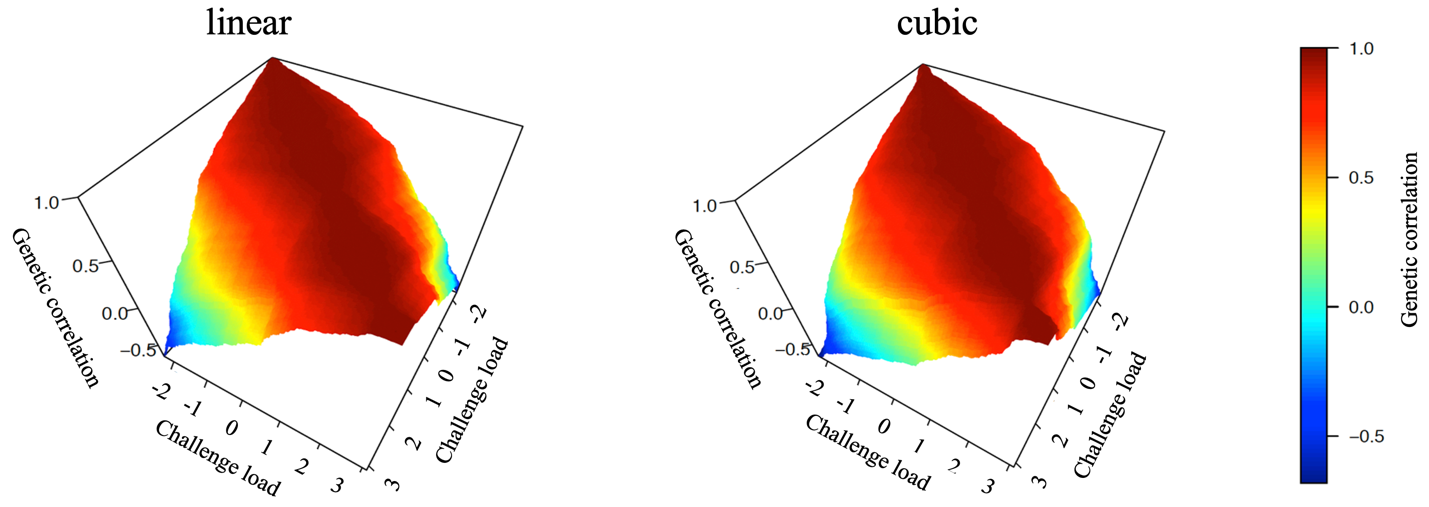


**Figure S1. Estimates of genetic correlations from the linear and the cubic spline reaction norm model for average daily gain (ADG, kg/d) in the challenge nursery using challenge load derived from early finisher growth rate.**


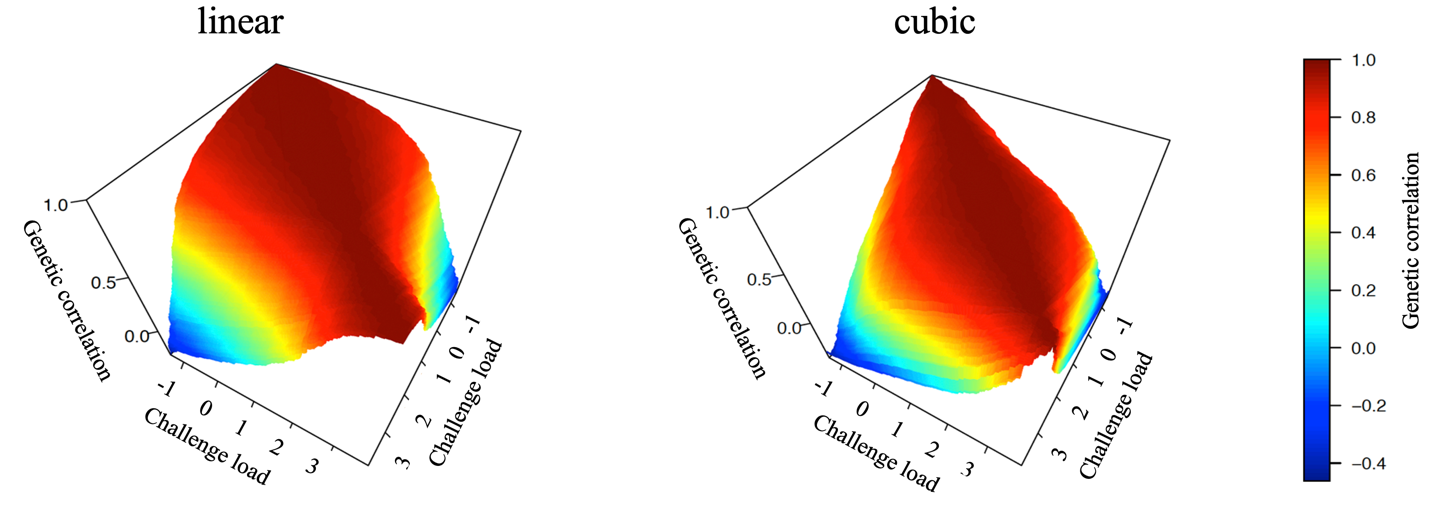


**Figure S2. Estimates of genetic correlations from the linear and the cubic spline reaction norm model for average daily gain (ADG, kg/d) in the finisher using challenge load derived from the clinical disease traits across the challenge nursery and finisher.**


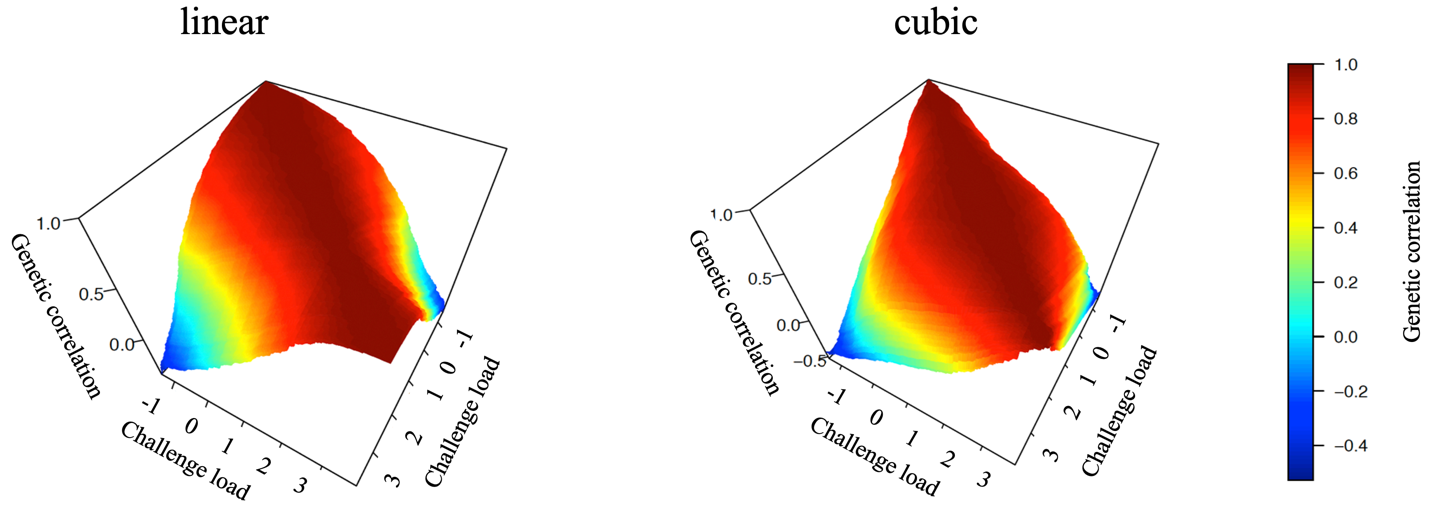


**Figure S3. Estimates of genetic correlations from the linear and the cubic spline reaction norm model for treatment rate in the challenge nursery using challenge load derived from the clinical disease traits across the challenge nursery and finisher.**


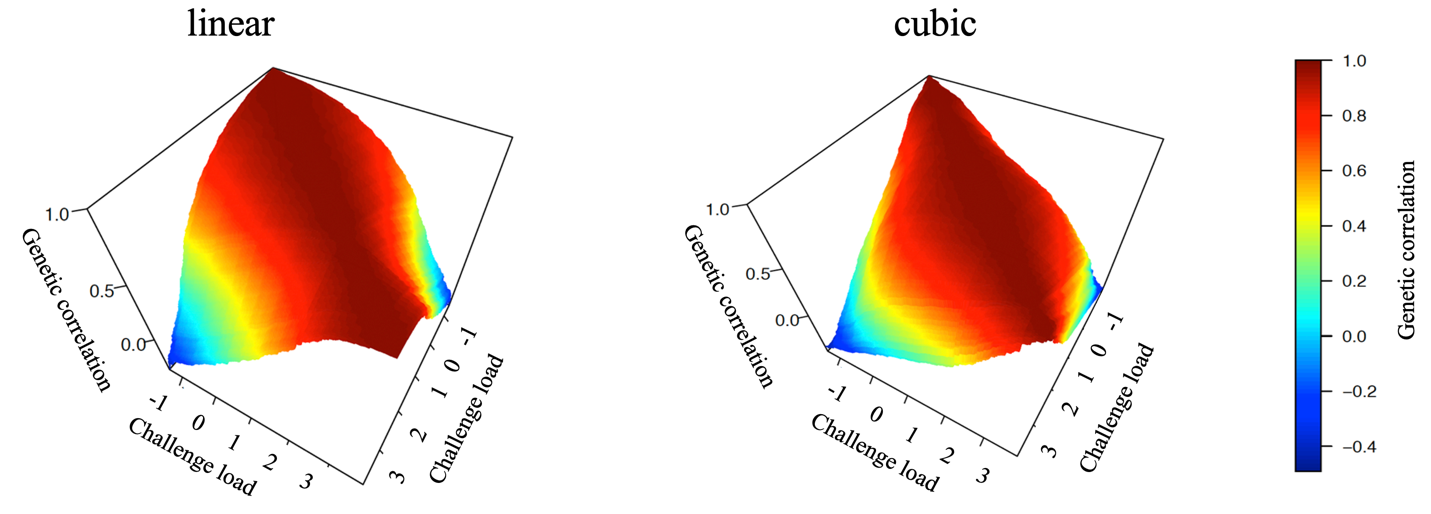


**Figure S4. Estimates of genetic correlations from the linear and the cubic spline reaction norm model for treatment rate across the challenge nursery and finisher using challenge load derived from the clinical disease traits across the challenge nursery and finisher.**

**Figure S5. Distribution and relationships of estimated breeding values for slope (including fixed effect estimate) from the cubic spline reaction norm model for average daily gain (ADG, kg/d) and treatment rate (TRT) in or across (combined) the challenge nursery and finisher.**

**Figure S6. Distribution and relationships of estimated breeding values for spline coefficient (including fixed effect estimate) from the cubic spline reaction norm model for average daily gain (ADG, kg/d) and treatment rate (TRT) in or across (combined) the challenge nursery and finisher.**

**Figure S7. Estimates of breeding values for four animals as a function of challenge load from the cubic spline reaction norm model for average daily gain (ADG, kg/d) and treatment rate (TRT) in or across (combined) the challenge nursery and finisher.**

Challenge load was derived using clinical disease traits across the challenge nursery and finisher
